# The human leukemic oncogene MLL-AF4 promotes hyperplastic growth of hematopoietic tissues in *Drosophila* larvae

**DOI:** 10.1016/j.isci.2023.107726

**Published:** 2023-08-25

**Authors:** Julie A. Johannessen, Miriam Formica, Aina Louise C. Haukeland, Nora Rojahn Bråthen, Amani Al Outa, Miriam Aarsund, Marc Therrien, Jorrit M. Enserink, Helene Knævelsrud

**Affiliations:** 1 Department of Molecular Cell Biology, Institute for Cancer Research, Oslo University Hospital, Oslo, Norway; 2 Centre for Cancer Cell Reprogramming, Institute of Clinical Medicine, Faculty of Medicine, University of Oslo, Oslo, Norway; 3 Department of Molecular Medicine, Institute of Basic Medical Sciences, Faculty of Medicine, University of Oslo, Oslo, Norway; 4 Institute for Research in Immunology and Cancer, Laboratory of Intracellular Signaling, Université de Montréal, C.P. 6128, Succursale Centre-Ville, Montréal, QC H3C 3J7, Canada; 5 Département de pathologie et de biologie cellulaire, Université de Montréal, Montréal, QC H3C 3J7, Canada; 6 Section for Biochemistry and Molecular Biology, The Department of Biosciences, Faculty of Mathematics and Natural Sciences, University of Oslo, Oslo, Norway

**Keywords:** Oncology, Molecular biology, Cell biology

## Abstract

*MLL*-rearranged (*MLL*-r) leukemias are among the leukemic subtypes with poorest survival, and treatment options have barely improved over the last decades. Despite increasing molecular understanding of the mechanisms behind these hematopoietic malignancies, this knowledge has had poor translation into the clinic. Here, we report a *Drosophila melanogaster* model system to explore the pathways affected in *MLL*-r leukemia. We show that expression of the human leukemic oncogene MLL-AF4 in the *Drosophila* hematopoietic system resulted in increased levels of circulating hemocytes and an enlargement of the larval hematopoietic organ, the lymph gland. Strikingly, depletion of *Drosophila* orthologs of known interactors of MLL-AF4, such as DOT1L, rescued the leukemic phenotype. In agreement, treatment with small-molecule inhibitors of DOT1L also prevented the MLL-AF4-induced leukemia-like phenotype. Taken together, this model provides an *in vivo* system to unravel the genetic interactors involved in leukemogenesis and offers a system for improved biological understanding of *MLL*-r leukemia.

## Highlights

• The human oncogene MLL-AF4 induces a leukemia-like phenotype in *Drosophila*
• Hematopoietic hyperplasia depends on homologs of known effectors in human leukemia
• The MEIS1/2/3 homolog hth is upregulated and required for MLL-AF4-induced hyperplasia
• The leukemic phenotype can be successfully inhibited with clinically relevant drugs

## Graphical abstract

**Figure:**
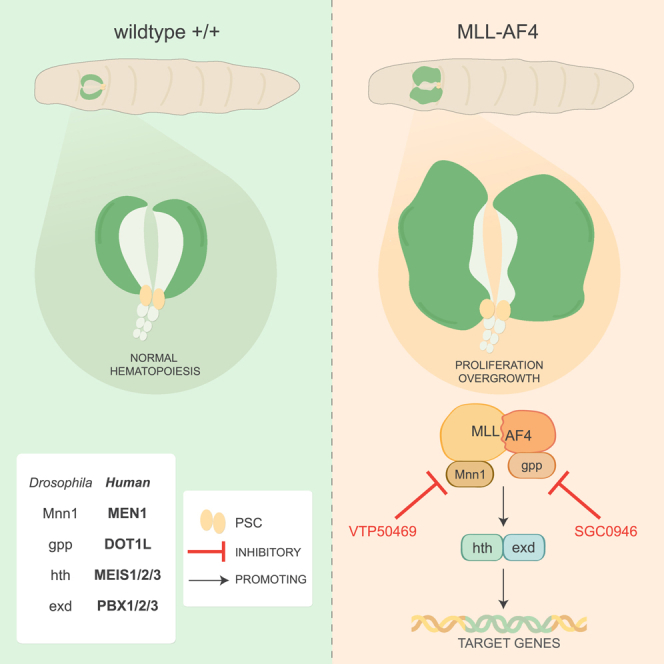

## Introduction

*Mixed Lineage Leukemia (MLL)*-rearranged leukemias are associated with dismal overall outcome, with 5-year overall survival rates as low as 8%–20% depending on the subtype.^1^^,^^2^ *MLL*-rearranged (*MLL*-r) leukemia is characterized by cytological alterations of chromosome 11q23, which were first identified in a wide range of patients with both myeloid and lymphocytic leukemia and later mapped to the *MLL* gene.^3^^,^^4^^,^^5^^,^^6^ The *MLL* gene encodes a methyltransferase and has therefore been renamed *Lysine [K]-specific Methyltransferase 2A* (*KMT2A*). Despite the efforts to improve and develop new treatment over the last 50 years, little progress has been made and patients diagnosed with *MLL*-r leukemia are left with a sparse range of therapy options and suffer poor survival rates.

Normal MLL positively regulates gene expression through histone modifications, directly as a histone 3 lysine 4 (H3K4) methyltransferase and indirectly by acting as an activator of the histone 4 lysine 16 (H3K16) acetyltransferase males absent on the first (MOF).^7^ Additionally, the MLL complex can also repress gene transcription dependent on CYP33-enabled recruitment of BMI1 and associated repressor proteins.^8^^,^^9^^,^^10^ Among the main target genes of normal MLL are *homeobox* (*Hox)* genes, which specify regions of the body plan through development and are involved in hematopoietic development,^11^ and *Meis* genes.^12^ Regulation of both sets of genes is aberrant in leukemic patients carrying *MLL* chromosomal rearrangement.^13^^,^^14^ In general, the N-terminal part of MLL is responsible for identifying and binding the target gene sequences. This occurs mainly through interaction with Menin, which recruits the H3K36 di/trimethylation reader lens epithelium-derived growth factor (LEDGF),^15^^,^^16^^,^^17^^,^^18^ and a CxxC domain, which binds to unmethylated CpG sequences.^19^^,^^20^ The C-terminal part of MLL contains the MOF interaction site and the SET domain responsible for the histone H3 lysine 4 methylation, associated with normal regulation of the target genes.^21^

In the MLL fusion proteins resulting from chromosomal translocations involving the *MLL* gene, the N-terminal part of MLL including the CxxC domain is retained, whereas the remainder of the protein is replaced by the C-terminal part of the fusion partner. To date, more than 100 such fusion partners have been described,^22^ but the most common ones represent about 80% of clinical cases (MLL fused with either *ALL1-fused gene* [AF] *from chromosome* 4 [AF_4_]/AFF1, eleven-nineteen-leukemia (ENL)/MLLT1, AF9/MLLT3, AF10/MLLT10 or eleven-nineteen lysine-rich leukemia (ELL)) and exist in related complexes involved in regulation of transcriptional elongation.^23^ For instance, the fusion partner AF4 is normally a nuclear protein involved in transcriptional activation,^24^ which directly interacts with the histone acetylation reader ENL.^25^ MLL-AF4 is the most frequent MLL translocation, predominantly in ALL,^22^ but the full molecular understanding of how this causes leukemia is still missing, including the importance of the reciprocal translocation AF4-MLL.^26^^,^^27^^,^^28^ The MLL-AF4 fusion protein is thought to drive a feedforward loop resulting in high activity of the histone H3 lysine 79 methyltransferase DOT1L, culminating in aberrant transcription of target genes.^23^ MLL-AF4 target genes include HOXA9, which has been shown to be dependent on its cofactors MEIS1 and PBX3 to induce leukemic transformation.^29^ *MEIS1* and *PBX3* are themselves also well-known MLL fusion protein targets.^30^^,^^31^ Even in the presence of this molecular understanding of the impact of MLL fusion proteins, therapies developed toward these targets have not yet successfully entered clinical practice. In this context, additional genetically tractable animal models that allow rapid and unbiased *in vivo* genetic analysis and biological understanding could complement studies in existing mouse models.

*Drosophila* has a long tradition as a system to understand gene function and to model disease, including cancer and leukemia.^32^ *Drosophila* hematopoiesis has several key similarities to mammalian hematopoiesis as it is governed by conserved transcriptional programs that result in generation of hemocytes with functional similarity to human blood cells.^33^ Fly immune cells reside in specific compartments where they interact with the microenvironment.^34^ Briefly, hematopoiesis in *Drosophila* occurs in two waves, first in the embryo and later in the larval hematopoietic organ, the lymph gland.^35^^,^^36^ The lymph gland consists of two primary (anterior) and several secondary (posterior) lobes. The primary lobe can be divided into the progenitor-containing medullary zone, the cortical zone containing differentiated hemocytes, and the posterior signaling center (PSC), which is involved in regulating the balance between progenitors and differentiated hemocytes.^37^ Traditionally, hematopoietic differentiation from prohemocytes has been known to result in three main types of hemocytes: plasmatocytes, crystal cells, and lamellocytes. The macrophage-like plasmatocytes make up the majority of hemocytes (around 95% of the hemocyte population) with a smaller fraction of crystal cells (around 5%) involved in melanization and wound healing.^38^ Finally, large, actin-rich lamellocytes develop upon specific insults, such as the presence of parasitic wasp eggs, and are not normally observed in healthy larvae.^39^^,^^40^ Recently, novel single-cell sequencing data have unraveled a more diverse range of hematopoietic cell subtypes, including intermediate stages.^41^^,^^42^ Furthermore, hemocyte development is not limited to the linear development from prohemocytes as a common precursor. There are also mechanisms of transdifferentiation, where plasmatocytes can develop into lamellocytes, underpinning the plasticity and intricacy of the *Drosophila* hematopoietic system.^43^

Notably, the MLL protein was first identified as Trithorax (Trx) in *Drosophila melanogaster*,^44^ and orthologs of all the main MLL fusion partners are found in *Drosophila*. Human full-length MLL was previously found to partly rescue a *trx* mutant. Interestingly, expression of human MLL-AF4 or MLL-AF9 in *Drosophila* larval brain affected cell-cycle progression, whereas ubiquitous expression was lethal.^45^ Furthermore, expression of human MLL-AF10 was found to deregulate the activity of *Polycomb* group-responsive elements in a *Drosophila* adult eye reporter system,^46^ indicating that these human leukemic fusion proteins are active also in *Drosophila*.

In this study, we exploited the conservation of the MLL complex and hematopoiesis in *D. melanogaster* to gain insight into the hematopoietic malignancies that occur upon chromosomal *MLL* rearrangements. We show that expression of human MLL-AF4 in the *Drosophila* hematopoietic system leads to increased levels of differentiated hemocytes, resulting in enlarged lymph glands and enhanced numbers of circulating hemocytes. These phenotypes were dependent on the main complex partners that have also been identified in human leukemia and could be rescued by genetic or chemical targeting of DOT1L.

## Results

### Expression of the human oncogene MLL-AF4 induces a leukemia-like phenotype in flies

**Figure 1:**
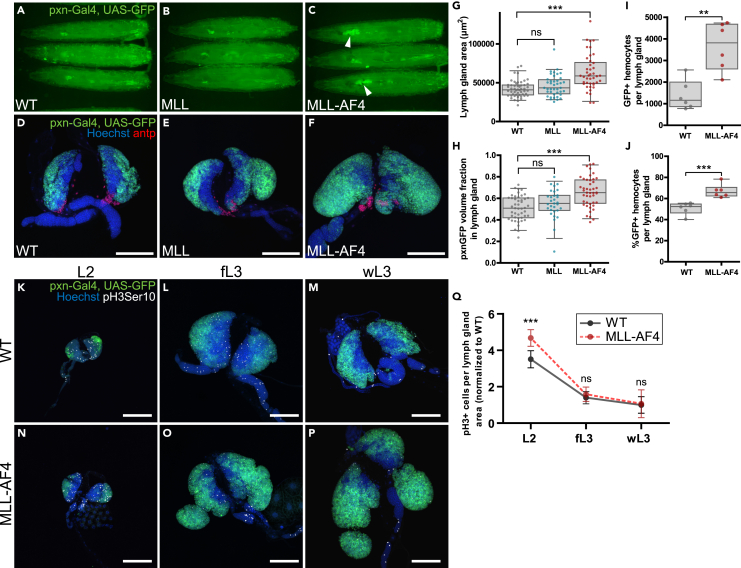
Expression of the human oncogene MLL-AF4 in the hematopoietic system of *D. melanogaster* induces a leukemia-like phenotype in the lymph gland (A–C) Representative images of wandering third-instar larvae expressing full-length human MLL or human MLL-AF4 driven by *pxn*-Gal4 and imaged by widefield fluorescence microscopy. The hematopoietic system is marked by GFP expression driven by *pxn*-Gal4. Arrowheads indicate enlarged lymph glands. (D–F) Immunofluorescence confocal images of lymph glands that are WT or expressing MLL or MLL-AF4 driven by *pxn*-Gal4. The cortical zone is marked by GFP, and the posterior signaling center (PSC) is detected by immunostaining against antp (in red). Images are maximum intensity projection of z stacks. Scale bars 100 μm. (G) Quantification of lymph gland area (μm^2^) from immunofluorescence confocal images. (H) Quantification of GFP-positive lymph gland volume presented as ratio of total lymph gland volume based on immunofluorescence confocal stacks. G, H: Each data point represents one animal. Boxplots show the mean ± the 95^th^ percentile as error bars. Statistical significance was determined by one-way ANOVA with Bonferroni post-test to assess significant differences from WT. ns: not significant, ∗∗∗: p < 0.001. (I) Quantification of GFP-positive hemocytes per lymph gland by flow cytometry. (J) Quantification of percentage of GFP-positive cells in larval lymph glands measured by flow cytometry. I, J: Data points are mean values from around 15 lymph glands per genotype from 6 independent experiments. Boxplots show the mean ± the 95^th^ percentile as error bars. Statistical significance was determined by two-sided Student’s *t* test to assess significant differences from WT. ∗∗: p < 0.01, ∗∗∗: p < 0.001. (K–P) Immunofluorescence confocal images of lymph glands from larvae that are WT or expressing MLL-AF4 driven by *pxn*-Gal4, in 2^nd^ instar larva (L2), feeding 3^rd^ instar larva (fL3) and wandering 3^rd^ instar larva (wL3), respectively. Cortical zone is marked by GFP expression and phospho-histone 3 Ser10 (pH3Ser10)-positive cells are shown in grayscale. Images are maximum intensity projection of z stacks. Scale bars 100 μm. (Q) Quantification of phospho-histone 3 Ser10-positive cells per lymph gland area for L2, fL3, and wL3 larval stages. All values are normalized to WT mean of wL3 larval stage. Values are shown as mean and error bars show 95^th^ percentile. Two-sided Student’s *t* test was performed to assess significant differences between WT and MLL-AF4 for each larval stage. ns: not significant, ∗∗∗: p < 0.001. Genotypes: A, D, G–J, K–M, Q: *pxn-Gal4, UAS-GFP/+* (WT) B, E, G–J: *pxn-Gal4, UAS-GFP/UAS-MLL* C, F, G–J, N–P, Q: *pxn-Gal4, UAS-GFP/UAS-MLL-AF4*. See also Figures S1 and S2.

Due to the conserved function of the equivalent of the human MLL complex in *Drosophila melanogaster*, the trithorax complex, we hypothesized that expressing the human oncogene MLL-AF4 in the hematopoietic system of *D. melanogaster* could induce a leukemic phenotype. We expressed either human full-length MLL or the human fusion protein MLL-AF4 in the larval hematopoietic system using the *pxn*-Gal4 driver and co-expressing GFP (*pxn*-Gal4>UAS-GFP). The *pxn*-Gal4 driver was chosen because *peroxidasin* is expressed in the cortical zone of the lymph gland from the second instar larval stage and onwards in hemocytes that mature into plasmatocytes and crystal cells.^36^^,^^47^ We assessed the overall effects of MLL-AF4 expression on the lymph gland by measuring lymph gland size changes. Furthermore, we evaluated the effects of MLL-AF4 expression on the cortical zone by quantification of the GFP+ volume fraction of the lymph gland, to identify whether this compartment changed in size relative to the overall lymph gland. Interestingly, while the lymph glands in larvae expressing full-length, normal MLL in the cortical zone were similar to wild-type (WT) lymph glands, expression of the MLL-AF4 oncogene induced significant lymph gland enlargement and an increase in GFP+ hemocytes in the lymph gland compared to WT larvae (Figures 1A–1G). Furthermore, lymph glands expressing MLL-AF4 in the cortical zone displayed increased levels of GFP volume per total lymph gland volume (Figure 1H). Flow cytometry of dissociated lymph glands also showed an increase in the total number of GFP+ hemocytes per lymph gland as well as an increase in the percentage of GFP+ hemocytes per lymph gland (Figures 1I and 1J), confirming that these lymph glands are hyperplastic. Lymph glands expressing either the MLL N-terminal or the AF4 C-terminal part of the MLL-AF4 fusion protein in the cortical zone were similar to controls (Figures S1A–S1F). We verified by qRT-PCR that the MLL N-terminal or the AF4 C-terminal portions were expressed to similar levels as the MLL-AF4 fusion protein (Figures S1G–S1I). We also found that simultaneous expression of two independent upstream activating sequence (UAS)-driven MLL-AF4 transgenes induced even larger lymph glands relative to a single MLL-AF4 transgene, bolstering the causal effect of MLL-AF4 expression on the hyperplasia phenotype (Figures S1J–S1L). However, larvae expressing MLL-AF4 were still able to develop into adult flies similar to the controls (Figure S1M).

To investigate whether the increase in lymph gland size was a result of increased proliferation, we stained lymph glands from L2, feeding L3 and wandering L3 stages for the proliferative marker phosphorylated histone 3 serine 10 (pH3Ser10). Indeed we found that the lymph gland overgrowth could be explained by increased proliferation in MLL-AF4-expressing lymph glands at the L2 larval stage, whereas there was no difference in proliferation in lymph glands from feeding and wandering L3 larvae (Figures 1K–1Q).

Given that *pxn-*Gal4 mainly drives MLL-AF4 expression in differentiating hemocytes,^36^ we determined whether its expression affected hemocyte maturation. The observed increase of GFP+ hemocytes was identified as plasmatocytes as shown by staining the primary lobes for NimC1/P1^48^ (Figures S2A–S2D). Furthermore, crystal cell levels were increased upon MLL-AF4 expression as evaluated by staining for Hindsight (Hnt)^49^ (Figures S2E–S2H). Finally, lamellocytes were occasionally observed in MLL-AF4-expressing lymph glands (Figures S2I–S2K). We also assessed the effect of cortical zone expression of MLL or MLL-AF4 on the PSC, which can be identified by antp-positive cells. The number of antp-positive cells was reduced upon expression of either MLL or MLL-AF4 (Figure S2L), suggesting that this was independent of the lymph gland hyperplasia induced by MLL-AF4 expression specifically.

**Figure 2:**
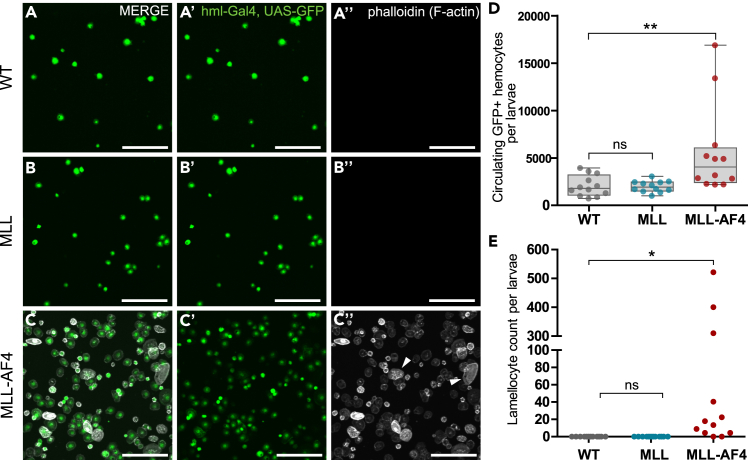
Expression of the human oncogene MLL-AF4 in the hematopoietic system of *D. melanogaster* induces a leukemia-like phenotype in circulating hemocytes (A–C) Immunofluorescence confocal images of circulating hemocytes from wL3 larvae that are WT or expressing MLL or MLL-AF4 driven by *hml*-Gal4. Images are maximum intensity projection of z stacks. Scale bars 100 μm. A′-C’: GFP+ cells marked by the driver *hml-*Gal4, UAS-GFP. A″-C’’: Phalloidin (F-actin) staining to visualize cytoskeleton and lamellocytes. Arrowheads indicate examples of differentiated lamellocytes. (D) Quantification of number of circulating hemocytes expressing GFP. Values are per larva across 4 larvae in 3 replicates. Boxplots show the mean ± the 95^th^ percentile as error bars. (E) Quantification of lamellocytes in circulating hemocytes from A–C. Note that the x axis is split between 100 and 300 to enable visualization of all values. D,E: One-way ANOVA with Bonferroni post-test was performed to assess significant differences. ns: not significant, ∗: p < 0.05, ∗∗: p < 0.01. Genotypes: A, D, E: *hml-Gal4, UAS-GFP/+* (WT) B, D, E: *hml-Gal4, UAS-GFP/UAS-MLL* C, D, E: *hml-Gal4, UAS-GFP/UAS-MLL-AF4*. See also Figure S3.

To further understand how MLL-AF4 affects the larval hematopoietic system, we investigated the effect of expressing MLL-AF4 in circulating hemocytes using the *hml*-Gal4 driver and co-expressing GFP (*hml*-Gal4>UAS-GFP).^50^ Whereas wL3 larvae expressing full-length MLL had normal levels of circulating hemocytes, MLL-AF4 expression resulted in a significant increase in circulating hemocyte levels (Figures 2A–2D). In L2 larvae the number of circulating hemocytes was similar to that of controls, indicating that the expansion occurs after this stage (Figures S3A–S3D). We also noticed that in larvae expressing MLL-AF4, lamellocytes were present in the hemolymph, again indicative of aberrant hemocyte differentiation (Figures 2A–2C and 2E). To test whether MLL-AF4-expressing hemocytes were still able to perform their phagocytic function, hemocytes were exposed to E. coli beads that fluoresce only at lower pH, indicative of internalization by phagocytosis. Larvae with hemocytes expressing either MLL or MLL-AF4 had similar levels of phagocytic cells compared to the control (Figure S3E), and these cells were performing phagocytosis at similar levels (Figure S3F). Taken together, these results show that MLL-AF4 induces hyperplasia of hematopoietic tissues in *Drosophila* primarily through an increase in plasmatocytes. In addition, MLL-AF4 expression affects hemocyte fate since it promotes the development of lamellocytes.

### Knockdown of MLL-AF4 complex partners rescues the leukemic phenotype

**Figure 3:**
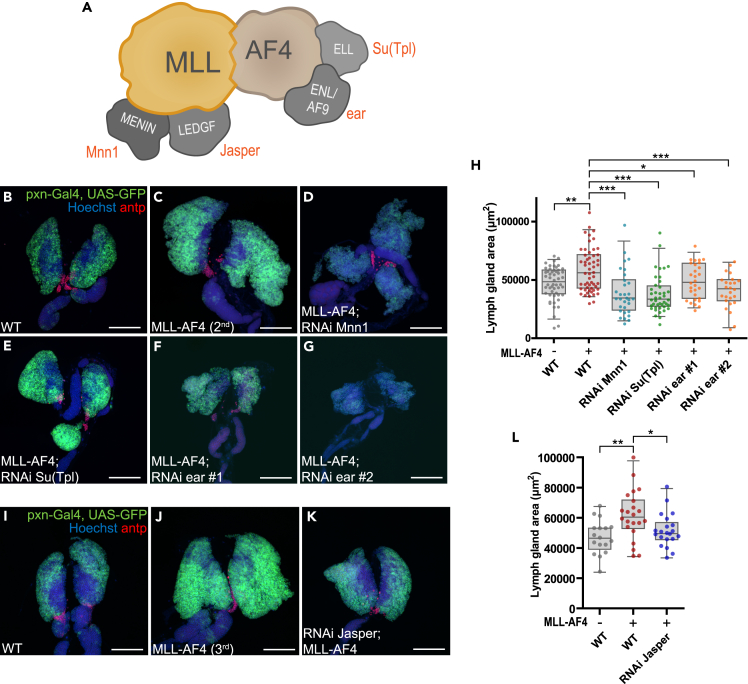
MLL-AF4-driven lymph gland enlargement depends on *Drosophila* homologs of known MLL-AF4 interaction partners (A) Schematic overview of some of the known direct interactors with the fusion protein MLL-AF4 in humans based on current literature. The corresponding *D. melanogaster* homologs are indicated in red. (B–G) and (I–K) Representative immunofluorescence confocal images of lymph glands with *pxn*-Gal4-driven expression of MLL-AF4 and concurrent depletion of indicated target genes (B–G: MLL-AF4 on the 2^nd^ chromosome, I–K: MLL-AF4 on the 3^rd^ chromosome). The cortical zone is marked by GFP expression and PSC is detected by immunostaining for antp (in red). Images are maximum intensity projection of z stacks. Scale bar 100 μm. (H and L) Quantification of lymph gland area from immunofluorescence images with MLL-AF4 expression driven by pxn-Gal4 combined with RNAi (H: MLL-AF4 on the 2^nd^ chromosome, M: MLL-AF4 on the 3^rd^ chromosome). H, L: Each data point represents one animal. Boxplots show the mean ± the 95^th^ percentile as error bars. One-way ANOVA with Bonferroni post-test was performed to assess significant differences from MLL-AF4. ∗: p < 0.05, ∗∗: p < 0.01, ∗∗∗: p < 0.001. Genotypes: B, H, I, L: *pxn-Gal4, UAS-GFP/+* (WT) C, H: *pxn-Gal4, UAS-GFP/UAS-MLL-AF4* D, H: *pxn-Gal4, UAS-GFP/UAS-MLL-AF4; UAS-RNAi Mnn1 GL00018/+* E, H: *pxn-Gal4, UAS-GFP/UAS-MLL-AF4; UAS-RNAi Su(Tpl) HMS00277/+* F, H: *pxn-Gal4, UAS-GFP/UAS-MLL-AF4; UAS-RNAi ear HMS00107/+* G, H: *pxn-Gal4, UAS-GFP/UAS-MLL-AF4; UAS-RNAi ear JF02905/+* J, L: *pxn-Gal4, UAS-GFP/+; UAS-MLL-AF4/+* K, L: *pxn-Gal4, UAS-GFP/UAS-RNAi Jasper HMC03961; UAS-MLL-AF4/+*. See also Figure S4.

Next, we tested whether the observed leukemia-like phenotypes depend on fly orthologs of the known complex partners of the human MLL-AF4 fusion protein (see Figure 3A for a schematic overview). To this end, we used RNAi-mediated knockdown of these orthologs. For MLL interaction partners we depleted Mnn1 (the *Drosophila* ortholog of MEN1) or Jasper (the *Drosophila* orthologue of LEDGF/PSIP1), since the human orthologs are required for MLL to interact with chromatin (Figure 3A). For interaction partners of human AF4, we targeted Su(Tpl) (the *Drosophila* ortholog of ELL) and ear (the *Drosophila* ortholog of ENL/MLLT1 and AF9/MLLT3), which are part of the transcriptional super elongation complex (SEC)^51^ (Figure 3A). Knockdown efficiency was evaluated by qRT-PCR in hemocytes for several available RNAi lines against each of these targets (Table S1; Figures S4A–S4D), and a subset of lines producing efficient knockdown was selected for phenotypic analysis. *Pxn*-driven depletion of most of these gene products in the cortical zone of otherwise WT lymph glands did not result in obvious changes in lymph gland size or morphology (Figures S4E–S4L). The exception was depletion of *Mnn1*, where lymph glands presented with larger secondary lobes than those in the WT, and the overall lymph gland size was smaller than that of WT (Figures S4G and S4L). Interestingly, in lymph glands expressing MLL-AF4 in the cortical zone, concurrent depletion of *Mnn1*, *Jasper*, *ear*, or *Su(Tpl)* resulted in a reduction in overall lymph gland size (Figures 3B–3L). Taken together, this shows that the observed leukemia-like phenotype of MLL-AF4 depends on the same conserved interaction partners as in human leukemia.

### The leukemic phenotype of the *Drosophila* MLL-AF4 model depends on the DOT1L fly homolog *gpp*

**Figure 4:**
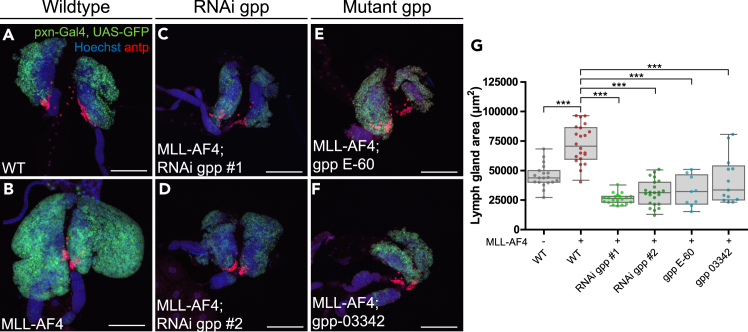
MLL-AF4-driven lymph gland enlargement depends on the *Drosophila* homolog of DOT1L, gpp (A–F) Representative immunofluorescence confocal images of lymph glands depleted of *gpp* in combination with MLL-AF4 expression driven by pxn-Gal4. The cortical zone is marked by GFP expression and PSC is detected by immunostaining for antp (in red). Images are maximum intensity projection of z stacks. Scale bars 100 μm. A, B: WT lymph gland and UAS-MLL-AF4-expressing lymph gland. C, D: MLL-AF4-expressing lymph glands combined with expression of dsRNA against *gpp*. E, F: MLL-AF4-expressing lymph glands from a heterozygous genetic background containing *gpp* mutants E−60 or 03342. (G) Quantification of lymph gland area (μm^2^) from immunofluorescence confocal images. Each data point represents one animal. Boxplots show the mean ± the 95^th^ percentile as error bars. One-way ANOVA with Bonferroni post-test was performed to assess significant differences from MLL-AF4. ∗∗∗: p < 0.001. Genotypes: A, G: *pxn-Gal4, UAS-GFP/+* (WT) B, G: *pxn-Gal4, UAS-GFP/UAS-MLL-AF4* C, G: *pxn-Gal4, UAS-GFP/UAS-MLL-AF4; UAS-RNAi gpp HMS00160/+* D, G: *pxn-Gal4, UAS-GFP/UAS-MLL-AF4; UAS-RNAi gpp JF1283/+* E, G: *pxn-Gal4, UAS-GFP/UAS-MLL-AF4; UAS-gpp E-60/+* F, G: *pxn-Gal4, UAS-GFP/UAS-MLL-AF4; UAS-gpp 03342/+*. See also Figure S5.

In *MLL*-r leukemia, the histone methyltransferase DOT1L has been shown to be associated with the MLL fusion protein complex, and DOT1L is required for leukemogenesis in different cellular and organismal model systems.^52^^,^^53^ Consequently, several clinical studies have focused on DOT1L as a potential therapeutic target, mainly in combination therapy with standard treatment.^54^^,^^55^^,^^56^ To test whether DOT1L is required for the MLL-AF4 phenotype in *Drosophila*, we first depleted the *Drosophila* DOT1L homolog *gpp*^57^ in lymph glands expressing MLL-AF4 in the cortical zone. Interestingly, *gpp* knockdown using two different RNAi lines resulted in complete rescue of the MLL-AF4-driven increase in lymph gland size (Figures 4A–4D and 4G). A similar rescue of the leukemic phenotype was also observed when MLL-AF4 was expressed in a heterozygous genetic background with a mutant allele of *gpp* (*gpp E-60* or *gpp-03342*) (Figures 4E–4G), in which *gpp* expression is reduced by half (Figure S5H). In contrast, in WT larvae, knockdown of *gpp* or introduction of *gpp* mutant alleles did not result in a significant reduction of lymph gland size (Figures S5A–S5F). Reduction of *gpp* expression in hemocytes by RNAi or mutant alleles was verified by qRT-PCR (Figures S5G and S5H). Altogether, these data show that *Drosophila* DOT1L is essential for the phenotype induced by human MLL-AF4 in this *in vivo* model system.

### The MLL-AF4 leukemia phenotype can be rescued by DOT1L inhibitors and MLL-Menin interaction inhibitors

**Figure 5:**
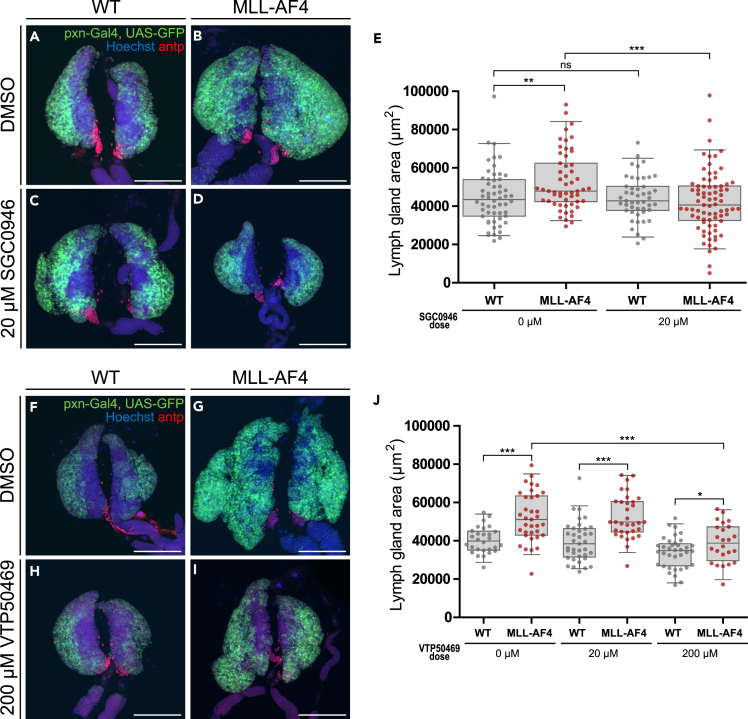
The MLL-AF4 *Drosophila* model is sensitive to drug treatment targeting DOT1L and the MLL-Menin interaction (A–D) and (F–I) Lymph glands from wandering 3^rd^ instar larvae. Cortical zone is marked by GFP expression and PSC is detected by immunostaining for antp (in red). Images are maximum intensity projection of z stacks. Scale bars 100 μm. A, B: WT and MLL-AF4-expressing lymph glands treated with DMSO only. C, D: WT and MLL-AF4-expressing lymph glands treated with 20 μM SGC0946. F, G: WT and MLL-AF4-expressing lymph glands treated with DMSO only. H, I: WT and MLL-AF4-expressing lymph glands treated with 200 μM VTP50469. (E) Quantification of lymph gland area (μm^2^) from immunofluorescence confocal images from drug treated larvae with either DMSO or the DOT1L inhibitor SGC0946. (J) Quantification of lymph gland area (μm^2^) from immunofluorescence confocal images from drug treated larvae with either DMSO or the MLL-Menin interaction inhibitor VTP50469. E, J: Each data point represents one animal. Boxplots show the mean ± the 95^th^ percentile as error bars. Statistical significance was determined by two-sided Student’s *t* test to assess significant differences between drug-treated larvae and DMSO control for each genotype. ns: not significant, ∗: p < 0.05, ∗∗: p < 0.01, ∗∗∗: p < 0.001.Genotypes: A, C, E, F, H, J: *pxn-Gal4, UAS-GFP/+* (WT) B, D, E, G, I, J: *pxn-Gal4, UAS-GFP/UAS-MLL-AF4*. See also Figure S6.

To understand if this model offers a reliable and rapid platform for identifying pharmacological inhibitors for human *MLL*-r leukemia, we next addressed whether drugs in preclinical and early-stage clinical studies are efficacious in the *Drosophila* MLL-AF4 leukemia model. We selected inhibitors targeting different downstream mechanisms of MLL-AF4. Specifically, we tested the inhibitor of MLL-menin interaction MI-463, the bromodomain and extraterminal motif inhibitor i-BET, and the DOT1L inhibitor SGC0946. Drugs of interest were administered to developing larvae by mixing them into the standard fly food. Drug effect on the hematopoietic system was assessed in wandering 3^rd^ instar larvae. Interestingly, initial experiments assessing drug effects by visualizing the hematopoietic system through the cuticle of intact larvae revealed that treatment with the DOT1L inhibitor seemingly reduced lymph gland size (Figures S6A–S6L), whereas MI-463 had little effect and i-BET induced larval death (Figures S6M–S6T). Therefore, we decided to investigate the DOT1L inhibitor effect further. Larvae were treated as before, and lymph glands were dissected out to assess drug effects on lymph gland size and morphology. Treatment with 20 μM SGC0946 was shown to fully rescue the lymph gland hyperplasia phenotype observed in the leukemia model (Figures 5A–5F). Notably, a subset of SGC0946-treated MLL-AF4-expressing lymph glands were even smaller than WT lymph glands, indicating that MLL-AF4-expressing hemocytes are particularly sensitive to inhibition of gpp/DOT1L.

Although DOT1L inhibitors showed promising results in initial studies, they have failed in clinical trials. To address whether the *Drosophila* MLL-AF4 leukemia model would respond to a more relevant novel therapy, we treated the larvae with the MLL-Menin interaction inhibitor VTP50469 that has showed potential in preclinical studies.^58^ Treatment with 20 μM showed no significant reduction in lymph gland size, whereas treatment with 200 μM, which was the limit concentration of solubility in DMSO, resulted in significantly smaller lymph glands in MLL-AF4-expressing larvae (Figures 5F–5J). Together, these data provide proof of principle that the MLL-AF4 *Drosophila* model is sensitive for pharmacological inhibition of clinically relevant targets that can be used to explore leukemogenesis.

### Hth and exd, the *Drosophila* homologs of MEIS1 and the PBX family, are crucial for the MLL-AF4 leukemic phenotype

**Figure 6:**
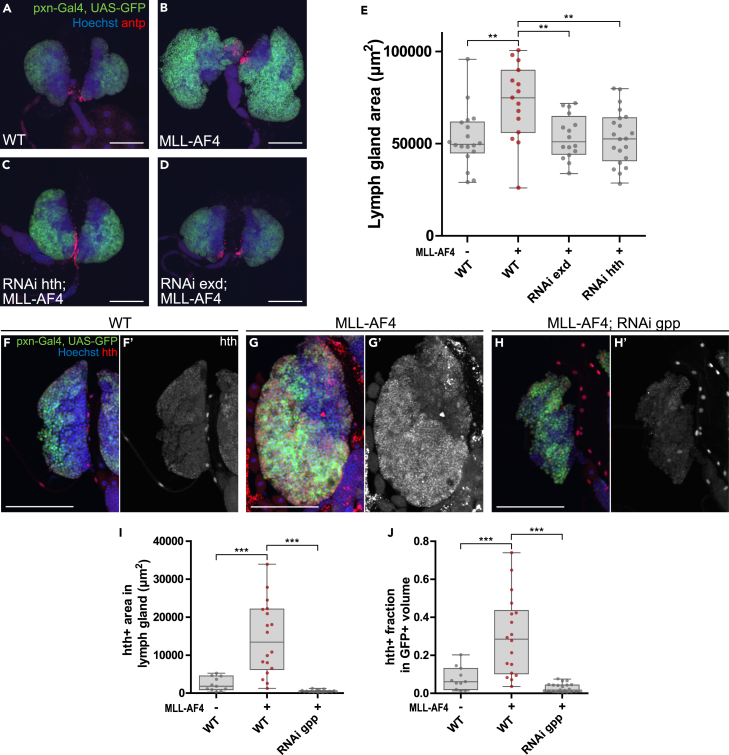
hth and exd, the *Drosophila* orthologs of MEIS1 and PBX, are crucial for the MLL-AF4 lymph gland enlargement phenotype (A–D) Lymph glands from wandering 3^rd^ instar larvae. Cortical zone is marked by GFP expression and PSC is detected by immunostaining for antp (in red). Images are maximum intensity projection of z stacks. Scale bars 100 μm. (E) Quantification of lymph gland area (μm^2^) from immunofluorescence confocal images. (F–H) Immunofluorescence confocal images of lymph glands stained for hth (red in merge, grayscale alone). (I) Quantification of hth-positive area in the lymph gland. (J) Quantification of the hth positive fraction of the cortical zone. E, I, J: Each data point represents one animal. Boxplots show the mean ± the 95^th^ percentile as error bars. One-way ANOVA with Bonferroni post-test was performed to assess significant differences from MLL-AF4. ∗∗: p < 0.01, ∗∗∗: p < 0.001. Genotypes: A, E, F, I, J: *pxn-Gal4, UAS-GFP/+* (WT) B, E: *pxn-Gal4, UAS-GFP/+; UAS-MLL-AF4/+* C, E: *pxn-Gal4, UAS-GFP/UAS-RNAi hth KK108831; UAS-MLL-AF4/+* D, E: *pxn-Gal4, UAS-GFP/UAS-RNAi exd KK107300; UAS-MLL-AF4/+* G, I, J: *pxn-Gal4, UAS-GFP/UAS-MLL-AF4* H, I, J: *pxn-Gal4, UAS-GFP/UAS-MLL-AF4; UAS-RNAi gpp HMS00160/+*. See also Figure S7.

*MLL*-r leukemia has previously been shown to depend on MEIS1 and co-depend on redundant contributions of PBX proteins in mammalian leukemia models.^30^^,^^59^ Furthermore, depletion of either of the *Drosophila* orthologs of MEIS1 or the PXB family, hth or exd, respectively, had previously been shown to rescue a HOXA9-NUP98 leukemia model in the *Drosophila* eye.^60^ To elucidate part of the biological mechanism leading to lymph gland hyperplasia in our MLL-AF4 leukemia model, we next investigated the role of hth and exd. Downregulation of either *hth* or *exd* rescued the MLL-AF4 lymph gland overgrowth (Figures 6A–6E), whereas it had no effect on WT lymph glands (Figures S7A–S7D). Furthermore, using immunostaining for hth, we could observe increased levels of hth-positive cells in the cortical zone of the lymph gland, where hth expression was highly upregulated in MLL-AF4-expressing hemocytes (Figures 6F–6I). The observed increase of hth-positive cells was not only a consequence of a larger cortical zone in MLL-AF4-expressing lymph glands, as shown by a higher fraction of hth expression also within the cortical zone (Figure 6J). Depletion of *gpp* reduced the hth signal back to basal levels, suggesting that the hth/exd axis is downstream of gpp recruitment and transcriptional activation.

## Discussion

*MLL*-r leukemia is associated with poor overall survival despite decades of studies, highlighting the need for *in vivo* models to identify actionable targets and novel compounds for treatment of this type of leukemia. Here, we characterized a *Drosophila* model, where expression of the human leukemic oncogene MLL-AF4 results in a leukemia-like phenotype that can be rescued by genetic or pharmacologic targeting of components required for leukemia development in mammalian model systems of *MLL*-r leukemia. This low-cost and rapid-to-screen model with the associated comprehensive toolbox for genetic manipulation and unbiased screening will allow for novel discoveries and sought-after therapeutic options *in vivo*.

In mouse models, DOT1L has been studied as a potential therapeutic target in *MLL*-r leukemia, using inhibitors that either inhibit its catalytic activity or disrupt its interaction with MLL fusion partners.^61^ Multiple mechanisms have been proposed to explain how the methyltransferase DOT1L contributes to leukemogenesis. Traditionally, it is believed that the fusion partner homologs AF9 and ENL are responsible for recruitment of DOT1L through the multi-subunit complex EAP (ENL-associated proteins), but DOT1L has also been shown to be directly associated with AF10.^62^ Furthermore, although MLL-AF4 does not appear to be directly associated with DOT1L, it can stimulate DOT1L activity indirectly through the recruitment of AF9.^63^^,^^64^ Pharmacological inhibition of DOT1L with small molecules selectively kills *MLL*-r leukemia cells *in vitro* and *in vivo.*^65^^,^^66^ In line with this, we found that a subset of MLL-AF4-expressing lymph glands from larvae raised on SGC0946-containing food were even smaller than WT lymph glands, indicating that MLL-AF4-expressing hemocytes are particularly sensitive to pharmacological gpp/DOT1L inhibition. Although SGC0946 effectively inhibits DOT1L activity, the failure of DOT1L inhibitors in clinical trials is concerning and may be attributed to the existence of compensatory mechanisms or alternative pathways that contribute to leukemogenesis. This fly model of leukemia could also be used as a convenient *in vivo* screening tool for identifying such mechanisms and pathways.

We also found genetic depletion of *gpp/DOT1L* to rescue the leukemia-like phenotype of our *MLL*-r leukemia model. Interestingly, the *gpp* allele E-60 was identified as an enhancer of the phenotype caused by expression of the human leukemic oncogene NUP98-HOXA9 in the *Drosophila* eye.^60^ In contrast, we found this allele to suppress the phenotype in our larval model of *MLL*-r leukemia, similar to the *gpp* allele 03342, which previously failed to complement lethal *gpp* alleles.^57^ Cells transformed by mechanistically distinct leukemic oncogenes, such as MLL-AF9, MLL-AF6, and NUP98-NSD1, have all shown sensitivity to DOT1L inhibition.^67^^,^^68^^,^^69^ As these oncogenes are known to activate *HOX* genes, DOT1L is probably promoting aberrant transcription of the *HOX* gene cluster in these malignancies. Interestingly, depletion of gpp in MLL-AF4-expressing lymph glands reduced the size to less than that in WT larvae, suggesting that they are unable to maintain their transcriptional regulation. These results are in concordance with our findings from the pharmacological inhibition of DOT1L where such sensitivity also was observed. When it comes to NUP98-HOXA9, the lack of gpp/DOT1L dependency might be due to the fusion oncoprotein containing the downstream target itself, hence bypassing the regulative effect of DOT1L on *HOX* genes. In this case, based on the *Drosophila* model,^60^ gpp/DOT1L appears to rather oppose NUP98-HOXA9 function, underscoring the need to critically evaluate the use of DOT1L inhibitors across cytogenetically different subtypes of leukemia.

Inhibitors of the BET family of proteins such as i-BET have shown promising results in preclinical models and in early clinical trials on hematologic malignancies,^70^ although clinical implementation of most of these inhibitors has been hampered by toxicity.^71^ Indeed, larvae feeding on food containing i-BET died at early larval stages irrespective of the presence of MLL-AF4, indicating general toxicity at concentrations above 5 μM. Therefore, we believe that our *Drosophila* model can be used to quickly discard candidate compounds or derivatives that are too toxic for further clinical development, which may help to reduce attrition commonly associated with drug development.^72^

Menin and LEDGF are other potential targets in *MLL*-r leukemia where small-molecule inhibitors have been developed.^73^^,^^74^^,^^75^ In agreement, genetic depletion of the *Drosophila* orthologs *Mnn1* and *Jasper* suppressed the lymph gland enlargement in this MLL-AF4 fly model. Lymph glands where Mnn1 was depleted in the presence of MLL-AF4 had a tendency to be smaller than WT on average, suggesting strong inhibition of the lymph gland growth in the absence of Mnn1. In contrast, the small molecule MI-463, which inhibits MEN1-MLL interaction, only produced a modest effect on the MLL-AF4-induced lymph gland hyperplasia. However, the novel MLL-Menin interaction inhibitor VTP50469 rescued the phenotype at high concentrations. VTP50469 has demonstrated significant promise as a therapeutic agent.^58^ Recently, additional novel Menin inhibitors have shown promising effects in clinical trials, underpinning the role of Menin as therapeutic target in *MLL*-r leukemia.^76^ The need to use high concentrations of VTP50469 in our model system could be due to limited bioavailability in the larvae, or that this compound is less efficient at disrupting the interaction of human MLL with *Drosophila* Mnn1.

Interestingly, we only observed a transient proliferative effect of MLL-AF4 in L2 larvae, whereas this effect was no longer present in wL3 larvae. It is possible that other cooperative factors are needed for a stronger phenotype and sustained proliferation, such as the reciprocal fusion protein AF4-MLL. Several model systems have failed to properly represent MLL-AF4-rearranged leukemia, and it has been proposed that this is due to the lack of the reciprocal fusion protein. Most patients with translocations of the MLL gene have also the reciprocal fusion protein present,^27^ and the reciprocal fusion protein has been shown to be necessary to establish a leukemic mouse model.^26^ A human infant model of *MLL*-r iALL, using CRISPR/Cas9 editing of human fetal cells to generate cells that express both MLL-AF4 and AF4-MLL, has also elucidated the importance of AF4-MLL.^77^ A future approach would be to co-express AF4-MLL in our fly leukemia model, preferably for a controlled duration since AF4-MLL may be lost during clonal evolution^27^ and may be most important at early disease development.^28^

Notably, in our model, depletion of hth or exd, the *Drosophila* orthologs of MEIS1 and PBX3, rescued the phenotype. These findings are in line with the role of MEIS1 and PBX3 in leukemic transformation in *MLL*-r leukemia previously described.^30^^,^^59^ In *Drosophila*, hth and exd have been shown to be associated with Hox proteins to regulate target developmental genes, a similar role of MEIS1 and PBX family.^78^ In the context of hematopoiesis, hth has been described in the regulation of the PSC, which is important for normal hematopoiesis. Specifically, *hth* mutant larvae fail to develop lymph glands and hth overexpression causes disappearance of the PSC.^79^^,^^80^ In agreement with this, we found that MLL-AF4 expression in the cortical zone lead to upregulation of hth protein levels and a decrease in the number of cells in the PSC. In contrast, the expression of NUP98-HOXA9 in the cortical zone induced an enlargement of the PSC,^81^ suggesting differences in the mechanisms of how these human oncogenes induce lymph gland hyperplasia. The fact that we do not see lymph gland development failure with *hth* knockdown is most likely because we restrict the expression pattern to mature hemocytes in our model, compared to the previously described hth null mutants. Since hth levels and lymph gland size could be rescued by gpp depletion, we suggest that hth and exd may function downstream of gpp recruitment and transcriptional activation. Future studies, such as by single-cell RNA sequencing, should investigate the changes in gene expression in this fly leukemia model dependent on gpp and hth/exd function, which may also explain the observed differentiation into lamellocytes. Interestingly, lamellocyte fate is often regarded as a suppressed cell fate and MLL-AF4 expression may relieve this suppression.^82^

Although previous *MLL*-r leukemia model systems have been reported in *D. melanogaster*, they have been limited to the observation of lethality, cell-cycle defects,^45^ or eye phenotypes in a reporter assay of *Polycomb* group-responsive element activity.^46^ These studies showed that expression of human MLL-AF4, MLL-AF9, or MLL-AF10 affected cell-cycle progression, normal development, or transcriptional regulation. In contrast, our viable *in vivo* model allows for a more detailed investigation of the hematopoietic irregularities that are induced upon MLL-AF4 expression. We observed changes in levels of differentiated hemocytes, specifically increased levels of plasmatocytes and crystal cells in the lymph gland as well as the presence of lamellocytes. These results are qualitatively more similar to those of the larval leukemia model based on NUP98-HOXA9 expression.^81^

In broader terms, *Drosophila* has previously been successfully employed to model leukemia-like phenotypes of other human leukemic oncogenes, such as AML1-ETO, NUP98-HOXA9, and BCR-ABL.^81^^,^^83^^,^^84^^,^^85^^,^^86^^,^^87^^,^^88^ In the context of BCR-ABL, a fly model of BCR-ABL expression in the fly eye has been put forward as a screening platform for identification of novel or improved compounds.^85^ In our study we found that the larval aberrant hematopoietic phenotype induced by human MLL-AF4 expression can be suppressed by DOT1L or Menin-MLL interaction inhibitors, indicating that our model could be used to test derivatives of current inhibitors or novel compounds. The phenotype is amenable to imaged-based screening, similar to what has previously been performed for a *Drosophila* Ras-driven tumor model,^89^ as the enlarged GFP-positive lymph gland can be visualized through the semi-transparent larval cuticle.

Collectively, we present a genetically tractable *MLL*-r leukemia model that is sensitive to pharmacological and genetic perturbations that can be exploited to further investigate the role of MLL-AF4 in leukemogenesis.

### Limitations of the study

Despite the valuable insights that can be gained from our fly leukemia model, we acknowledge the limitations of our system. There are several limitations when it comes to using this for drug screening in terms of scaling and pharmacokinetics. Based on an imaging approach, it is likely not realistic to use this platform for thousands of compounds. Moreover, actual drug concentrations in the larvae are not easily measured. It is possible to implement feeding assays to control and assess dosage of the drugs in each individual larva, but this will not be feasible for large-scale drug screens. Furthermore, we have not evaluated the potential degradation of drugs when mixed in the fly food. This could potentially lead to false negatives, and one should assess the pharmacological properties of the drug before exposing it to fly food. The drug platform serves better for proof-of-concept studies in the context of *MLL*-r leukemic pathways.

Both the DOT1L inhibitor SGC0946 and the MLL-Menin interaction inhibitor VTP50496 rescued the MLL-AF4-induced lymph gland enlargement. One significant limitation is that our model system could not accurately predict which of the two drugs would exhibit favorable outcomes in clinical trials. The failure of DOT1L inhibitors in clinical trials minimizes its role as a therapeutic candidate, whereas the potential of MLL-Menin interaction and targeted Menin inhibitors as agents is highly promising.^58^^,^^76^ Findings observed in our model system may not necessarily translate directly to the clinical setting due to complex biological variations between *Drosophila* and humans.

Although we observe hematopoietic aberrations in *Drosophila* upon the expression of MLL-AF4, our model differs from human leukemia in some important aspects. Whereas accumulation of immature blasts is observed in leukemia resulting from *MLL*-r leukemia in humans, we do not observe an accumulation of immature hemocytes in our model. Based on the available assays and markers, the increase in hemocyte numbers in circulation and in the cortical zone of the lymph gland appears to represent terminally differentiated and functional hemocytes.

This model only includes MLL-AF4 and not the reciprocal translocation AF4-MLL. AF4-MLL may be essential to sustain the initial increase in proliferation (pSer10His3 staining) that we observe in L2 lymph glands, similar to a suggested role in human *MLL*-r leukemia.^28^ There might be leukemic transformations that are not properly modeled due to the lack of this and other important genetic factors. However, despite its limitation as a drug candidate screening system, our model system holds great potential for understanding MLL biology in the context of hematopoiesis and leukemia. By interrogating the underlying molecular mechanisms and drug responses, our model system can aid in the discovery of new targets and guide the development of more effective treatments for leukemia.

## STAR★Methods

### Key resources table

**Table:** 

REAGENT or RESOURCE	SOURCE	IDENTIFIER
**Antibodies**		

mouse anti-Hnt	DSHB	Cat# 1g9, RRID:AB_528278
mouse anti-Antp	DSHB	Cat# anti-Antp 4C3, RRID:AB_528082
mouse anti-NimC1/P1	I. Ando^48^	RRID:AB_2568423
mouse anti-Attila/L1	I. Ando^90^	N/A
mouse anti-phospho-Histone H3Ser10	Abcam	Cat# ab14955, RRID:AB_443110
goat anti-hth	Santa Cruz	Cat# sc-26187, dG20 RRID:AB_672050
donkey anti-mouse Alexa568	Molecular Probes	Cat# A10037, RRID:AB_2534013
donkey anti-rat DyLight649	Jackson Immunoresearch	Cat#712-495-153, Lot#93224, RRID:AB_2560938
donkey anti-goat Alexa555	Molecular Probes	Cat# A-21432, RRID:AB_2535853
mouse anti-Wg	DSHB	Cat# 4d4, RRID:AB_528512

**Chemicals, peptides, and recombinant proteins**		

pHrodo Red *E. coli* BioParticles	Invitrogen	Cat# P35361, Lot# 1387791
Alexa Fluor 647 Phalloidin	Molecular Probes	Cat#A22287

**Experimental models: Organisms/strains**		

w, pxn-Gal4, UAS-GFP	F. Lemieux^91^	N/A
w^iso^	M.Therrien	N/A
w; UAS-MLL-AF4 ML5 (on 2^nd^)	R. Paro^45^	N/A
w; UAS-MLL-AF4 ML6 (on 3^rd^)	R. Paro^45^	N/A
w; UAS-MLL FL on 2nd	R. Paro^45^	N/A
w; UAS-MLL N-term (on 3^rd^)	R. Paro^45^	N/A
w; UAS-MLL N-term (on X)	R. Paro^45^	N/A
w; UAS-AF4 C-term on X	R. Paro^45^	N/A
UAS-RNAi ear TRiP.HMS00107}attP2	BDSC	RRID:BDSC_34798
UAS-RNAi ear TRiP.JF02905}attP2	BDSC	RRID:BDSC_28068
UAS-RNAi Su(Tpl) TRiP.GL00168}attP2	BDSC	RRID:BDSC_35270
UAS-RNAi Su(Tpl) TRiP.HMS00277}attP2	BDSC	RRID:BDSC_33399
UAS-RNAi Mnn1 TRiP.HMC03436}attP40	BDSC	RRID:BDSC_51862
UAS-RNAi Mnn1 TRiP.JF01775}attP2	BDSC	RRID:BDSC_31220
UAS-RNAi Mnn1 TRiP.GL00018}attP2	BDSC	RRID:BDSC_35150
[1118]; UAS-RNAi Mnn1 GD8117	VDRC	17701
UAS-RNAi Mnn1 KK101050	VDRC	110376
UAS-RNAI-CG7946 TRiP.HMC03961}attP40	BDSC	RRID:BDSC_55274
y[1] v[1]; UAS-gpp-RNAi (TRiP.JF01284) (on 3rd)	BDSC	RRID:BDSC_31327
y[1] v[1]; UAS-gpp-RNAi (TRiP.JF01283) (on 3rd)	BDSC	RRID:BDSC_31481
UAS-RNAi gpp VDRC 47199	VDRC	47199
y[1] sc[∗] v[1]; UAS-RNAi-gpp (TRiP.HMS00160) (on 3rd)	BDSC	RRID:BDSC_34842
y[1] sc[∗] v[1]; UAS-gpp-RNAi (TRiP.GL01325) (on 3rd)	BDSC	RRID:BDSC_41893
y[1] sc[∗] v[1]; UAS-gpp-RNAi (TRiP.HMS02612) (on 2nd)	BDSC	RRID:BDSC_42919
UAS-gpp-RNAi KK107875	VDRC	110264
gpp E-60/TM6b	M. Therrien^60^	N/A
gpp[03342]/TM6b	BDSC	RRID:BDSC_11585
y[1] v[1]; UAS-luciferase-RNAi TRiP.JF01355	BDSC	RRID:BDSC_31603
w[1118]; P{w[+mC]=UAS-lacZ.NZ)J312	BDSC	RRID:BDSC_3956
UAS-RNAi-lilli TRiP.JF02087}attP2	BDSC	RRID:BDSC_26314
UAS-RNAi-lilli TRiP.HMS01066}attP2	BDSC	RRID:BDSC_34592
UAS-hth-RNAi KK108831	VDRC	100630
UAS-exd-RNAi KK107300	VDRC	100687

**Oligonucleotides**		

qRT-PCR primer sets	See Table S2	N/A

**Software and algorithms**		

FlowJo	Becton, Dickinson & Company	RRID:SCR_008520
GraphPad Prism	GraphPad Software	RRID:SCR_002798
NIS Elements	Nikon	RRID:SCR_014329
Adobe Photoshop	Adobe	RRID:SCR_014199
FIJI		RRID:SCR_002285
FACSDiva	BD	RRID:SCR_001456
StepOne software	Applied Biosystems	RRID:SCR_014281

### Resource availability

#### Lead contact

Further information and requests for resources and reagents should be directed to and will be fulfilled by the lead contact, Helene Knævelsrud (helene.knavelsrud@medisin.uio.no).

#### Materials availability

This study did not generate new unique reagents. All fly lines used in this manuscript are either available from stock centers or will be made available upon request.

### Experimental model and study participant details

#### Fly stocks

*Drosophila melanogaster* fly stocks were mainly obtained from Bloomington Stock Center or VDRC. *UAS-MLL-AF4* ML5 (on 2nd), *UAS-MLL-AF4* ML6 (on 3rd), *UAS-MLL* FL on 2^nd^, *UAS-MLL* FL on 3^rd^, *UAS-MLL N-term* (on 3rd), *UAS-MLL N-term* (on X) and *UAS-AF4 C-term* (on X) were reported elsewhere.^45^ pxn-Gal4, UAS-GFP was also previously reported.^91^ A full list of the fly lines used in this study can be found in Table S1. All crosses were performed on standard potato mash fly food (27.3 g/liter dry yeast, 32.7 g/liter dried potato powder, 60 g/liter sucrose, 0.73% agar, 0.2% 4-hydroxybenzoic acid methyl ester (nipagin)/ethanol and 0.45% propionic acid) with additional yeast paste and incubated at 25°C for the first 72 hrs followed by 48-72 hrs at 29°C to boost Gal4-driven expression.

### Method details

#### GFP imaging of entire larvae

Larvae were heat fixed to visualize the lymph gland in intact animals. Wandering L3 larvae were collected, washed once in PBS with 0.2% Triton-X100 (Sigma Aldrich, Cat#T9485) (PBT 0.2%) and once in PBS, followed by heat fixation in a drop of glycerol on a microscope slide on a heat block set at 70°C for approximately 10 seconds. A coverslip was added on top of the glycerol drop with the heat fixed larvae before imaging them with a GFP filter on Leica MZFLIII stereomicroscope and LAS v4.9 software. Post-imaging enhancement of GFP signal for sufficient visualization was performed in Adobe Photoshop.

#### Immunofluorescence confocal microscopy of lymph glands

For dissection of lymph glands, larvae were washed once in PBT 0.2% and once in PBS 1x. Lymph glands were dissected in PBS 1x together with the mouth hook and CNS to simplify washing and staining. The tissue was fixed with 4% paraformaldehyde (Polysciences, Cat# 18814-20) for 10 min, and then washed in PBT 0.2% three times. Samples were then blocked in PBT 0.2% with 5% bovine serum albumin (Roche, Cat# 10735094001) before staining with primary antibodies overnight at 4°C. The primary antibodies used in this study were Hnt (1:10; DSHB 1G9, deposited by H.D. Lipshitz), Antp (1:100; DSHB 4C3, deposited by D. Brower), NimC1/P1 (1:300; I. Ando^48^), Attila/L1 (1:100; I. Ando^90^), phospho-Histone H3Ser10 (1:500; Abcam ab14955), hth (1:50, Santa Cruz, Cat# sc-26187). For staining with the Attila/L1 antibody, Triton-X100 was omitted in all washing, blocking and staining steps and replaced with PBS 1X to ensure proper staining. Lymph glands were next washed twice with PBT 0.2% and then incubated with fluorescent secondary antibodies for 2 hours in room temperature in the dark, followed by 10 min incubation with 1μg/mL Hoechst-33342 (Thermo Scientific™, Cat# 62249, Lot# PK1922313). The secondary antibodies used in this study were anti-mouse Alexa568 (1:1000, Molecular Probes, Cat# A10037), anti-rat DyLight649 (1:1000, Jackson Immunoresearch, Cat#712-495-153, Lot#93224) and anti-goat Alexa555 (1:500, Molecular Probes, Cat# A21432). After staining, lymph glands were washed twice in PBT 0.2%, detached from the carcass and mounted on microscope slides with 15 μL ProLong™ Diamond Antifade Mountant (Invitrogen™, Cat#P36961). Typically, between 10 and 25 lymph glands were imaged and analyzed per genotype. Confocal imaging was performed on a Nikon Yokogawa CSU-W1 with a S Plan-FI LWD 20x air objective and a laser set of 405 nm, 488 nm and 561 nm. Lymph glands were imaged in a z-stack covering 25 μm with a z-step of 0.1 μm.

#### Immunofluorescence of circulating hemocytes

For confocal imaging of circulating hemocytes, larvae were bled in ice-cold ESF921 media (Expression Systems, Cat# 500302) and the hemolymph from one larva was transferred to a corresponding well in a black 384-well glass-bottom plate (CellVis, Cat# P384-1.5H-N) The circulating hemocytes were fixed with 4% paraformaldehyde (Polysciences, Cat# 18814-20) and centrifuged for 5 min at 1000 rpm before incubation for 10 min at room temperature. Wells were then washed twice with PBT 0.2% and blocked with PBT 0.2% containing 5% bovine serum albumin (Roche, Cat# 10735094001). Primary antibody staining with anti-Wg (1:500, DSHB 4D4, deposited by S. M. Cohen) was done for 1 hour at room temperature, followed by 3 washes of PBT 0.2%. Wells were incubated with Alexa Fluor™ 647 Phalloidin (1:100, Molecular Probes, Cat#A22287) together with secondary antibody anti-mouse Alexa Fluor™ 568 (1:1000, Molecular Probes, Cat# A10037) for 1 hour at room temperature and washed once in PBS. Circulating hemocytes from wL3 larvae were mounted with 15 μL ProLong™ Diamond Antifade Mountant (Invitrogen™, Cat#P36961) per well, whereas circulating hemocytes from L2 larvae were mounted with 15 μL of Ibidi Mounting Media with DAPI (Ibidi, Cat# 50011, Lot# 22-05-02). Imaging was done with Nikon Yokogawa CSU-W1 with S Plan-FI LWD 20x air objective where 5 images per larva was acquired.

#### qRT-PCR

qRT-PCR was used to determine RNA levels of genes of interest, as well as to validate knockdown efficiency of RNAi fly lines. For qPCR on circulating hemocytes, the hemocytes were extracted by a protocol adapted from Tattikota and Perrimon.^92^ For each biological replicate, 15-20 larvae were collected in a 2 mL Eppendorf tube containing 500 μl glass beads and 200 μl PBS. The tube was vortexed for 2 min at 2000 rpm. Only larvae where the heartbeat was detectable were included in the following. Each larva was dried and placed in a 10μl drop of ice-cold PBS before peeling the cuticle open allowing the hemocytes to enter the media by diffusion. The next larva was added to the same drop and the procedure repeated until all larvae were processed before the hemolymph was transferred to a new tube. RNA was extracted using TRIzol (Life Technologies, Cat#15596026, Lot#16200701) and the Zymo Research Direct-zol™ RNA MicroPrep kit (Cat# R2062, Lot# ZRC201718) and RNA concentration measured by NanoDrop. RNA was converted to cDNA using the iScript™ cDNA Synthesis Kit (BIO-RAD Cat#1708891) on the Applied Biosystems SimpliAmp PCR machine. qRT-PCR was performed with Fast SYBRGreen Master Mix (Applied Biosystems, Cat#4385614) and run on Applied Biosystems StepOnePlus Real Time PCR system with StepOne v2.3 software. Efficiency of primer sets was tested before running expression level analysis. qPCR primers were selected from http://www.flyrnai.org/flyprimerbank.^93^ All primers used are listed in Table S2.

#### Flow cytometry of live lymph gland cells

Flow cytometry was used to determine lymph gland size and differentiation through levels of mature GFP-positive hemocytes in the lymph gland. Around 15 lymph glands were dissected out and trypsinized with 100 μL of 1x trypsin with EDTA without phenol red (Life Technologies™, Cat#15400054) for 15 minutes at 25°C. Each sample was pipetted up and down 40 times to ensure proper tissue dissociation. The reaction was quenched with 300 μL of PBS with 2% FBS, before the sample was filtrated in FACS tubes with 35 μm filter cap (Falcon®, Cat# 352235). 1 μL of 1,677 mg/mL Propidium Iodide (Sigma Aldrich, Cat# P4170) was added right before analysis to identify live and dead cells. Samples were run on LSRII UV laser flow cytometer and the analysis was performed with FACSDiva and FlowJo software.

#### Phagocytosis assay of circulating hemocytes

To assess if the circulating hemocytes expressing MLL-AF4 were functional, we measured phagocytic activity with flow cytometry. Hemolymph from 15 wandering third instar larvae was collected by bleeding in 100 μL ice-cold ESF921 (Expression Systems, Cat# 500302) and kept on ice after collection. Cells were counted using a Bürker chamber and equal number of cells were mixed with 1, 2.5 and 5 μL pHrodo™ Red *E. coli* BioParticles™ (Invitrogen, Cat# P35361, Lot# 1387791) for 20 minutes at room temperature. 300 μL PBS with 2% FBS was added before the sample was filtrated in FACS tubes with 35 μm filter cap (Falcon®, Cat# 352235). Samples were run on LSRII UV laser flow cytometer and the analysis was performed with FACSDiva and FlowJo software.

#### Drug treatment

SGC0946 (Selleckchem, Cat# S7079 and MedChemtronica Cat# HY-15650/CS-3531, Lot#58947), VTP50469 (MedChemExpress, Cat# HY-114162, Lot# CS-0077527), i-BET (Merck Millipore, Cat# 401010) and MI-463 (Selleckchem, Cat# S7816) were dissolved in DMSO as a stock solutions and added to molten (∼40°C) standard fly food mixed with blue food colorant (Panduro, Cat# 1018108-104) at final concentrations of the drug and 0.1% DMSO. DMSO concentration was kept equal across all experimental vials. The exception was for the preliminary drug experiments in Figure S6, where DMSO concentration varied and DMSO only controls were provided for each drug concentration. 5 mL of food was added to each vial and allowed to solidify. Crosses were initially performed on normal fly food with yeast paste before they were flipped onto vials with drugs 24 hours after crossing to ensure sufficient fertilization and egg laying. Incubation times and temperature conditions were as in regular crosses described above. The presence of blue color in the larval gut was used as an indication to ensure exposure to the drug by ingestion of the drug-containing food.

### Quantification and statistical analysis

#### Image processing and quantification

Within each set of experiments, images were captured with identical settings below pixel value saturation and post-processed identically. Intensity enhancements for visualization purposes on confocal microscopy images were performed using Adobe Photoshop, ImageJ or NIS Elements, and all intensity alterations were done equally for all genotypes to ensure comparativeness within each experiment. Confocal images shown in figures are maximum intensity projections made using the NIS Elements software unless otherwise stated. For confocal imaging performed on the Nikon Yokogawa CSU-W1 microscope, lymph gland volume and cell type volume fractions were calculated using the NIS Elements software. Quantifications for circulating hemocytes, lamellocytes, NimC1/P1+ cells, Hnt+ cells, antp+ cells and hth+ area were also performed with NIS Elements. NimC1/P1 positive cells were counted in 1 Z-plane visualizing the surface plane only due to improper antibody penetration of lymph gland tissue. Phospho-H3 positive cell quantifications were done using ImageJ and is reported per lymph gland area to account for size differences.

#### Experimental design and statistics

Samples were not masked or randomized during data collection or analysis. Statistical analysis was performed using GraphPad Prism 5. The data was assumed to be normally distributed. For multiple comparisons, one-way ANOVA with Bonferroni post-testing was used. For single comparisons, unpaired two-sided Student’s t-test was performed. For each panel, the employed statistical test is stated in the corresponding figure legend.

## Data Availability

• Microscopy, flow cytometry and qPCR data reported in this paper will be shared by the lead contact upon request. • ImageJ macros and NIS Elements software setting used for quantification will be shared by the lead contact upon request. • Any additional information required to reanalyze the data reported in this paper is available from the lead contact upon request.
