# Supplementary material for: The human leukemic oncogene MLL-AF4 promotes hyperplastic growth of hematopoietic tissues in Drosophila larvae
Source: iScience. 2023 Aug 25;26(10):107726. doi: 10.1016/j.isci.2023.107726 (PMC10504488; doi:10.1016/j.isci.2023.107726)
Supplement: Document S1. Figures S1–S7 and Tables S1–S3 [file mmc1.pdf]

iScience, Volume *26*

Supplemental information

The human leukemic oncogene MLL-AF4 promotes hyperplastic growth of hematopoietic tissues in *Drosophila* larvae

Julie A. Johannessen, Miriam Formica, Aina Louise C. Haukeland, Nora Rojahn Bråthen, Amani Al Outa, Miriam Aarsund, Marc Therrien, Jorrit M. Enserink, and Helene Knævelsrud

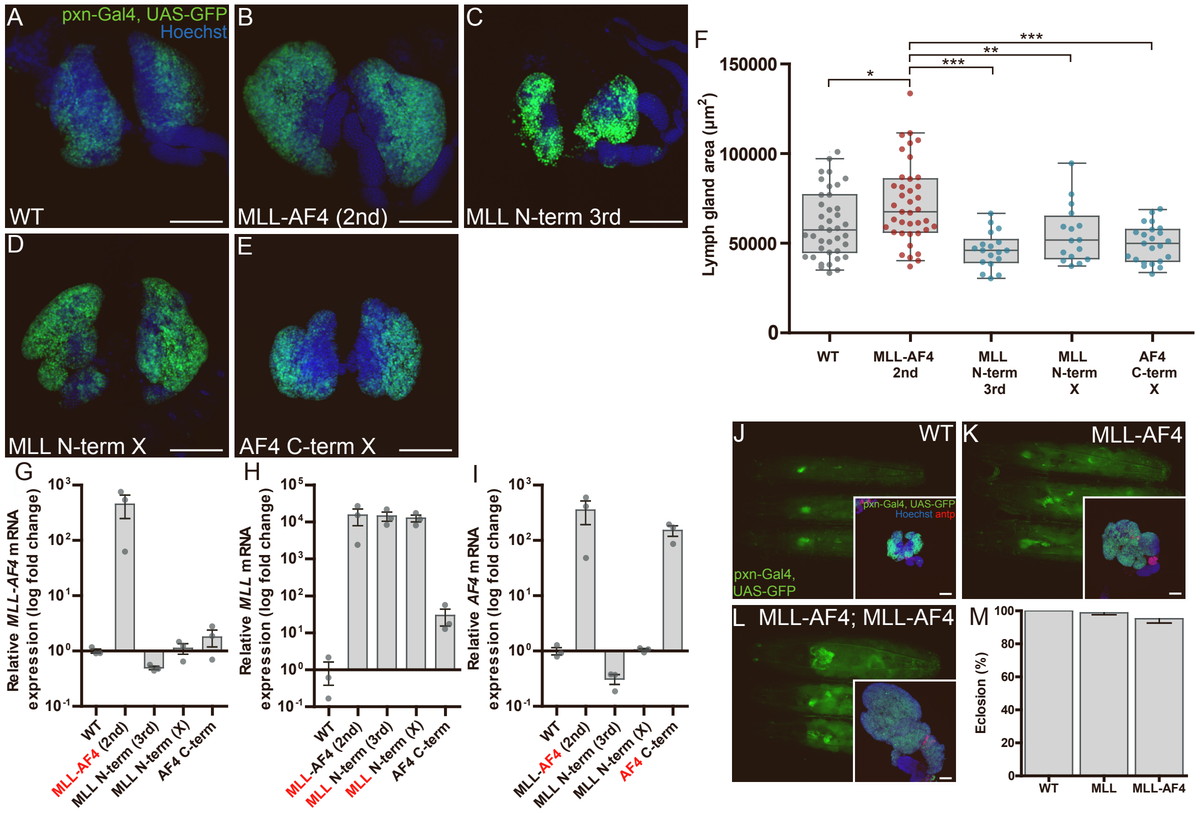

Figure S1: Expression of the N-terminal part of MLL or the C-terminal part of AF4 does not induce lymph gland hyperplasia, related to Figure 1. A-E: Immunofluorescence confocal images of lymph glands expressing human MLL-AF4, the N-terminal part or the C-terminal part of the MLL-AF4 transgene in the cortical zone. Cortical zone is marked by GFP expression. For MLL N-term, images are shown for transgenes on the 3rd and the X chromosome. Images are maximum intensity projection of Z-stacks. Scale bars 100 µm. **F**: Quantification of lymph gland area (µm^2) from immunofluorescence confocal images of lymph glands expressing either MLL-AF4 transgenes, the N-terminal MLL part or the C-terminal AF4 part of the fusion protein. Each data point represents one animal. Box plots show the mean ± the 95th percentile as error bars. One-way ANOVA with Bonferroni post-test was performed to assess significant differences. *: $p<0.05$, **: $p<0.01$, ***: p<0,001 **G-I**: Expression levels of MLL-AF4 transgenes as measured by qRT-PCR. The part of the transgene that is detected by the qPCR primer set is highlighted in red. Experiments were conducted in three biological replicates and in technical duplicates, and values are normalized to relative expression in WT larvae. Bars show mean values from replicates. Error bars show standard error of the mean (SEM). **A-I:** 2nd, 3rd and X refers to chromosome of transgene loci. **J-K:** Representative images of whole wandering third instar larvae expressing full-length human MLL, human MLL-AF4 on 2nd chromosome or larvae expressing MLL-AF4 transgenes on both 2nd and 3rd chromosome driven by *pxn*-Gal4 imaged by widefield fluorescence microscopy. Inserts: Representative immunofluorescence confocal images of lymph glands. Scale bar 100 µm. **M:** Percentage of pupa that result in eclosion of adult flies. Values are average of eclosion percentage from 5 biological replicates per genotype, where number of larvae per replicate cross varied from 5 to 45. Error bars shows standard error of the mean (SEM). **Genotypes: A, F, J, M**: *pxn-Gal4, UAS-GFP/+* (WT) **B, F, K, M**: *pxn-Gal4, UAS-GFP/UAS-MLL-AF4* **C, F:** *pxn-Gal4, UAS-GFP/+;UAS-MLL-N-term/+* **D, F***:* *UAS-MLL-N-term/+; pxn-Gal4, UAS-GFP/+* **E, F:** *UAS-AF4 C-term/+; pxn-Gal4, UAS-GFP/+* **G-I:** *hml-Gal4, UAS-GFP/+* (WT). *hml-Gal4, UAS-GFP/UAS-MLL-AF4* (UAS-MLL-AF4 (2nd)). *Hml-Gal4, UAS-GFP/+;UAS-MLL-N-term/+* (UAS-MLL N-term (3rd)). *UAS-MLL-N-term/+; hml-Gal4, UAS-GFP/+* (UAS-MLL N-term (X)). *UAS-AF4 C-term/+; hml-Gal4, UAS-GFP/+* **L**: *pxn-Gal4, UAS-GFP/UAS-MLL-AF4; UAS-MLL-AF4/+* **M**: *pxn-Gal4, UAS-GFP/UAS-MLL*

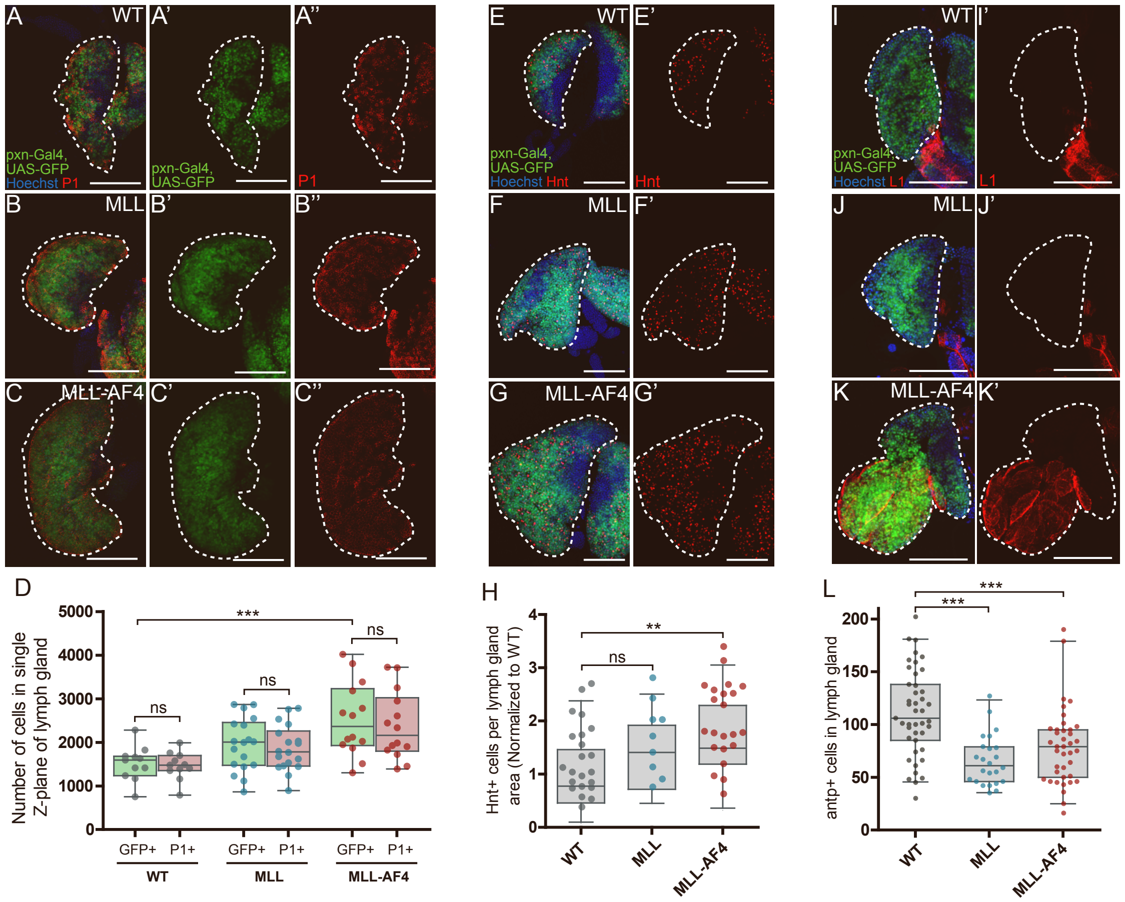

Figure S2: Characterization of the effects of MLL-AF4 on different hemocyte cell types, related to Figure 1. A-C: Immunofluorescence confocal images of lymph glands. The cortical zone is marked by GFP expression driven by *pxn*-Gal4 (A'-C') and plasmatocytes are detected by staining against NimC1/P1 (shown in red, A''-C''). One primary lobe is outlined per genotype. **D:** Quantification of P1+ cells per GFP+ cell in lymph gland from immunofluorescence confocal images in A-C. **E- G:** Immunofluorescence confocal images of lymph glands. Cortical zone is marked by GFP expression driven by *pxn*-Gal4 and crystal cells are detected by staining against Hnt (shown in red, E'-G'). One primary lobe is outlined per genotype. Scale bars 100 µm. **H:** Quantification of Hnt+ cells per lymph gland from immunofluorescence confocal images in E-G. **I-K:** Immunofluorescence confocal images of lymph glands. Cortical zone is marked by GFP expression driven by *pxn*-Gal4 and lamellocytes are detected by staining against L1 (shown in red, I'-K'). One primary lobe is outlined per genotype. Scale bars 100 µm. **L:** Quantification of antp+ cells per lymph gland from immunofluorescence confocal images from 3 replicate experiments. **D, H, L:** Each data point represents one animal. Box plots show the mean ± the 95th percentile as error bars. One-way ANOVA with Bonferroni post-test was performed to assess significant differences. ns: not significant, **: p<0.01, ***: p<0,001. **Genotypes: A, D, E, H, I, L:** *pxn-Gal4, UAS-GFP/+* (WT) **B, D, F, H, J, L**: *pxn-Gal4, UAS-GFP/UAS-MLL* **C, F, G, H, K, L:** *pxn-Gal4, UAS-GFP/UAS-MLL-AF4*

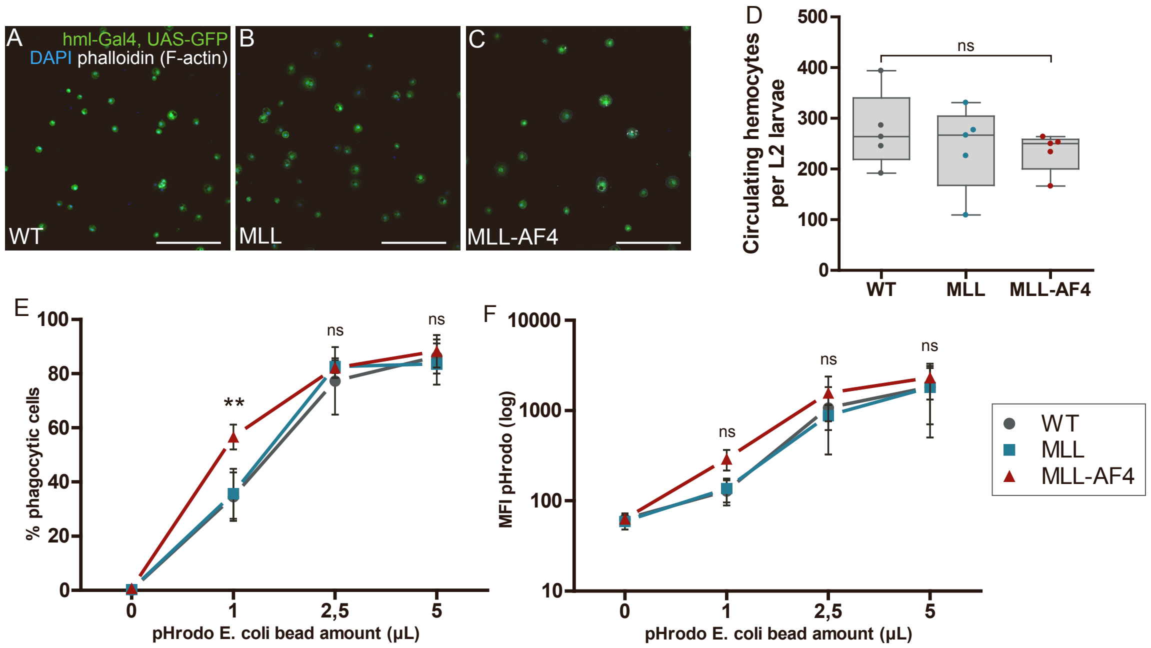

Figure S3: The increased number of circulating hemocytes consists of differentiated mature hemocytes and lamellocytes, related to Figure 2. A-C: Immunofluorescence confocal images of circulating hemocytes from L2 larvae that are WT or expressing MLL or MLL-AF4 driven by *hml*-Gal4. Images are maximum intensity projection of Z-stacks. Scale bars 100 µm. **D:** Quantification of number of circulating hemocytes expressing GFP. Each data point represents average numbers for 3 larva, shown for 5 replicates. Box plots show the mean ± the 95th percentile as error bars. One-way ANOVA with Bonferroni post-test was performed to assess significant differences. ns: not significant. **E:** Quantification of the percentage of phagocytic cells based on flow cytometry measurement of uptake of pHrodo E. coli beads in hemocytes. **F:** Quantification of the mean fluorescence intensity (MFI) of pHrodo signal from E. coli beads phagocytosed in hemocytes. **E, F:** Values shown are average for 3 replicate experiments. Error bars show standard error of the mean (SEM)**.** One-way ANOVA with Bonferroni post-test was performed to assess significant differences for each amount of beads. ns: not significant, **: p<0.01. **Genotypes: A, D-F:** *hml-Gal4, UAS-GFP/+* (WT) **B, D-F:** *hml-Gal4, UAS-GFP/UAS-MLL* **C, D-F:** *hml-Gal4, UAS-GFP/UAS-MLL-AF4*

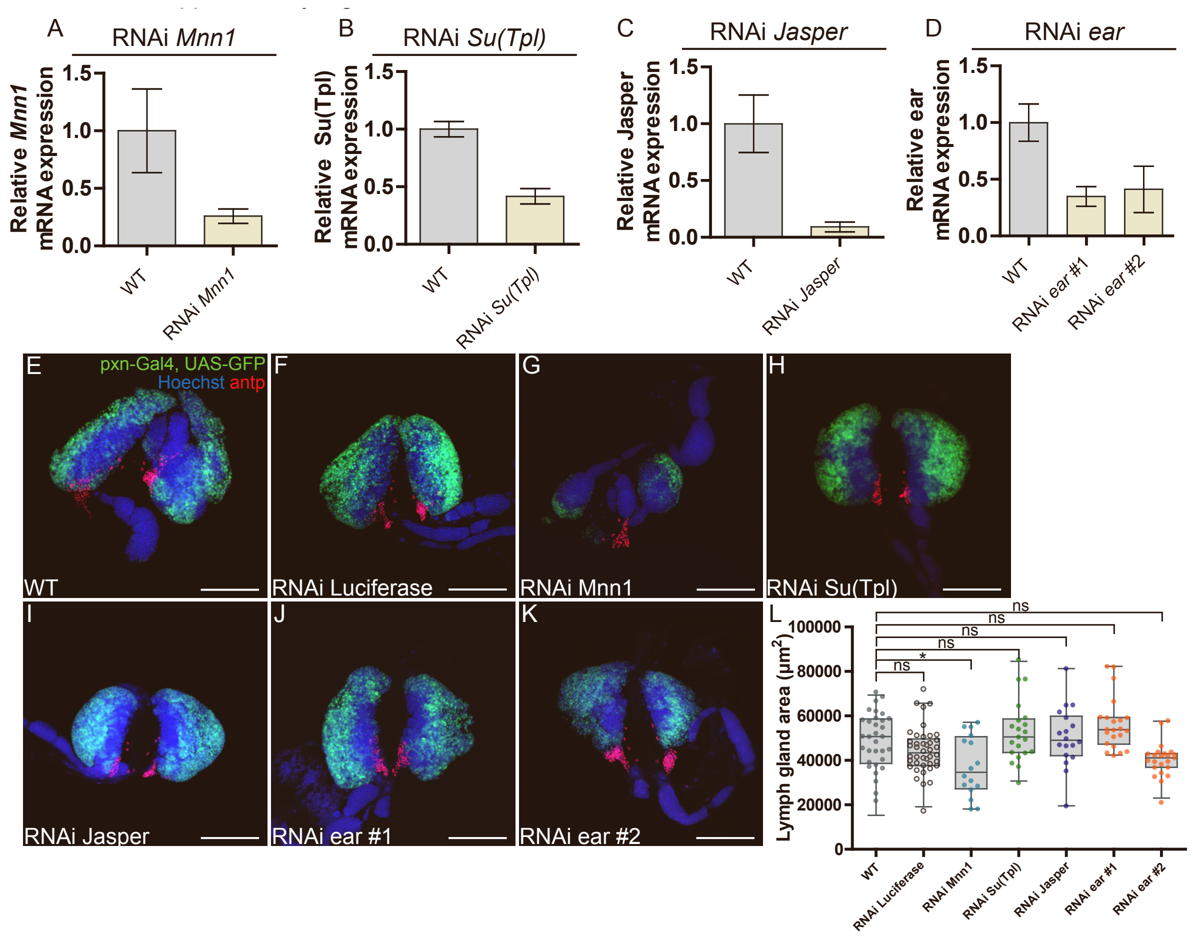

Figure S4: Depletion of MLL-AF4 complex partners alone does not affect lymph gland size, related to Figure 3. A-D: Validation of knock-down efficiency through qRT-PCR measurements of relative expression of target genes. Experiments were conducted in three biological replicates and in technical duplicates, and values are normalized to relative expression in WT larvae. Bars show mean values from replicates. Error bars show the standard error of the mean (SEM). Complete overview over qRT-PCR data for RNAi lines can be found in Table S3. **E-K:** Representative immunofluorescence confocal images of lymph glands expressing RNAi targeting *Mnn1*, *Su(Tpl)*, *ear* and *Jasper* driven by *pxn*-Gal4. Cortical zone is marked by GFP expression and PSC is detected by immunostaining for antp (in red). Images are maximum intensity projection of Z-stacks. Scale bars 100 µm. **L:** Quantification of lymph gland area (µm^2) from immunofluorescence confocal images of RNAi lines shown in E-K. Each data point represents one animal. Box plots show the mean ± the 95th percentile as error bars. One-way ANOVA with Bonferroni post-test was performed to assess significant differences. ns: not significant, *: $p<0.05$. **Genotypes: A-D:** *hml-Gal4, UAS-GFP/+* (WT) **A:** *hml-Gal4, UAS-GFP/UAS-MLL-AF4; UAS-RNAi Mnn1 GL00018/+* **B:** *hml-Gal4, UAS-GFP/UAS-MLL-AF4; UAS-RNAi Su(Tpl) HMS00277/+* **C:** *hml-Gal4, UAS-GFP/UAS-RNAi Jasper HMC03961; UAS-MLL-AF4/+* **D**: *hml-Gal4, UAS-GFP/UAS-MLL-AF4; UAS-RNAi ear HMS00107/+* (#1) and *hml-Gal4, UAS-GFP/UAS-MLL-AF4; UAS-RNAi ear JF02905/+* (#2) **E, L:** *pxn-Gal4, UAS-GFP/+* (WT) **F, L:** *pxn-Gal4, UAS-GFP/+; UAS-RNAi Luciferase/+* **G, L**: *pxn-Gal4, UAS-GFP/UAS-MLL-AF4; UAS-RNAi Mnn1 GL00018/+* **H, L:** *pxn-Gal4, UAS-GFP/UAS-MLL-AF4; UAS-RNAi Su(Tpl) HMS00277/+* **I, L:** *pxn-Gal4, UAS-GFP/UAS-RNAi Jasper HMC03961; UAS-MLL-AF4/+* **J, L**: *pxn-Gal4, UAS-GFP/UAS-MLL-AF4; UAS-RNAi ear HMS00107/+* (#1) **K, L**: *pxn-Gal4, UAS-GFP/UAS-MLL-AF4; UAS-RNAi ear JF02905/+* (#2)

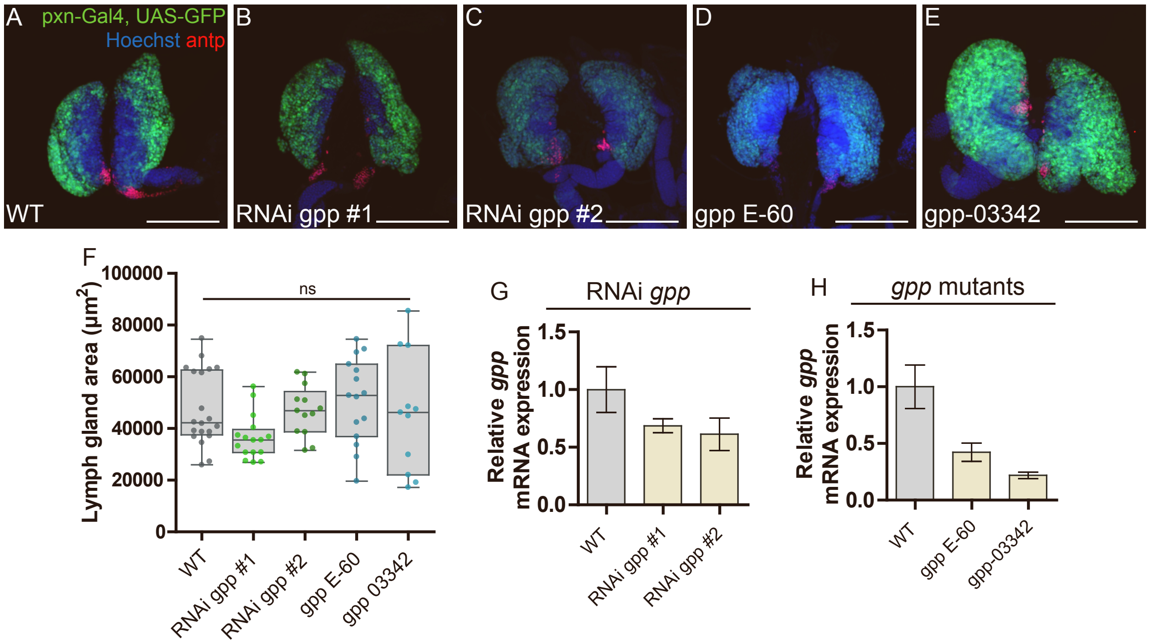

Figure S5: *gpp* RNAi and *gpp* mutant alleles do not affect the lymph gland phenotype, related to Figure 4. A-E: Representative immunofluorescence confocal images of lymph glands with reduced *gpp* levels through RNAi driven by *pxn*-Gal4 or introduction of *gpp* mutant alleles. Cortical zone is marked by GFP expression and PSC is detected by immunostaining for antp (in red). Images are maximum intensity projection of Z-stacks. Scale bars 100 µm. **F:** Quantification of lymph gland area (µm^2) from immunofluorescence confocal images in A-E. Box plots show mean ± the 95th percentile as error bars. One-way ANOVA with Bonferroni post-test was performed to assess significant differences. ns: not significant. **G, H:** Validation of knock-down through qRT-PCR measurements of relative *gpp* expression levels in circulating hemocytes for RNAi lines and *gpp* alleles, respectively. Experiments were conducted in three biological replicates and in technical duplicates, and values are normalized to relative expression in WT larvae. Bars show mean values from replicates. Error bars show the standard error of the mean (SEM). Complete overview over qRT-PCR data for RNAi lines can be found in Table S3. **Genotypes: A, F:** *pxn-Gal4, UAS-GFP/+* (WT) **B, F:** *pxn-Gal4, UAS-GFP/+; UAS-RNAi gpp HMS00160/+* (#1) **C, F:** *pxn-Gal4, UAS-GFP/+; UAS-RNAi gpp JF1283/+* (#2) **D, F:** *pxn-Gal4, UAS-GFP/+; UAS-gpp E-60/+* **E, F:** *pxn-Gal4, UAS-GFP/+; UAS-gpp 03342/+* **G, H:** *hml-Gal4, UAS-GFP/+* (WT) **G:** *hml-Gal4, UAS-GFP/+; UAS-RNAi gpp HMS00160/+* (#1) and *hml-Gal4, UAS-GFP/+; UAS-RNAi gpp JF1283/+* (#2) **H:** *hml-Gal4, UAS-GFP/+; UAS-gpp E-60/+* and *hml-Gal4, UAS-GFP/+; UAS-gpp 03342/+*

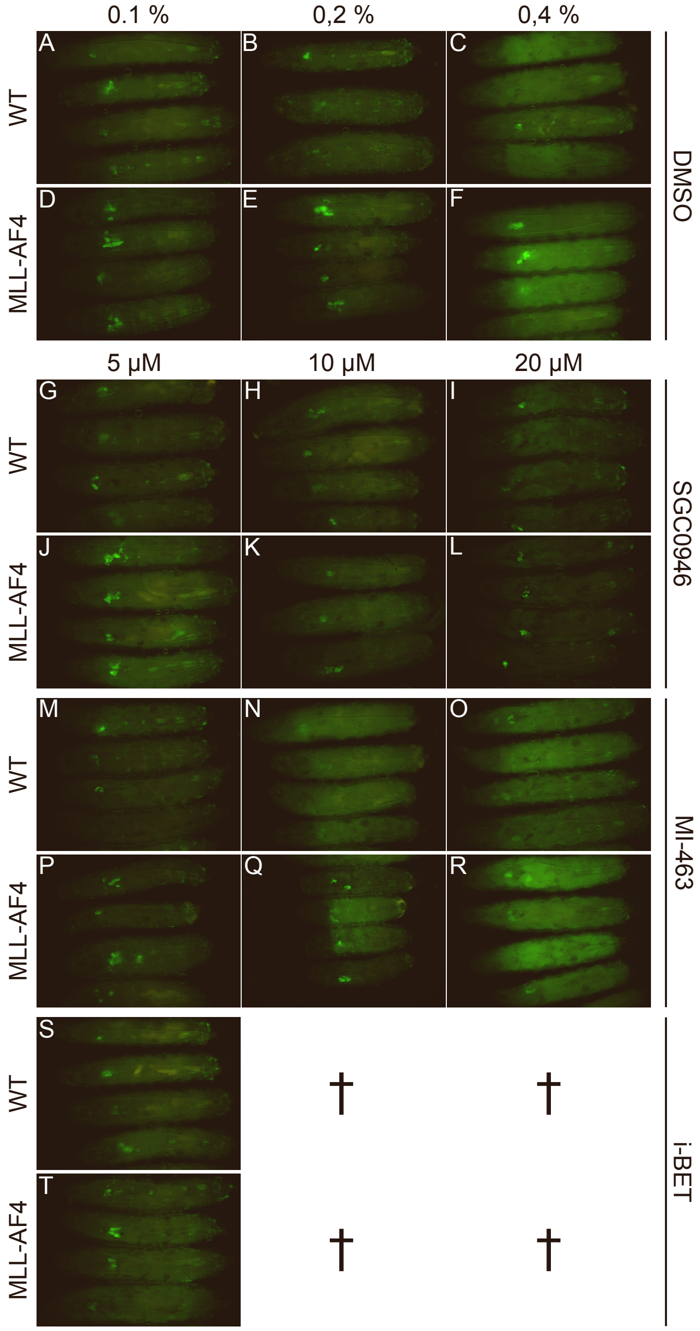

Figure S6: Drug treatment of larvae expressing MLL-AF4, related to Figure 5. A: Representative images of whole wandering third instar larvae treated with SGC0946, MI-643 and i-BET respectively in doses 5, 10 and 20 µM imaged by widefield fluorescence microscopy. Larvae are either WT or expressing human MLL-AF4 driven by *pxn*-GFP. The hematopoietic system is marked by GFP expression (*pxn*-Gal4). †: No live larvae after drug treatment. **Genotypes: A-C, G-I, M-O, S:** *pxn-Gal4, UAS-GFP/+* (WT) **D-F, J-L, P-R, T:** *pxn-Gal4, UAS-GFP/UAS-MLL-AF4*

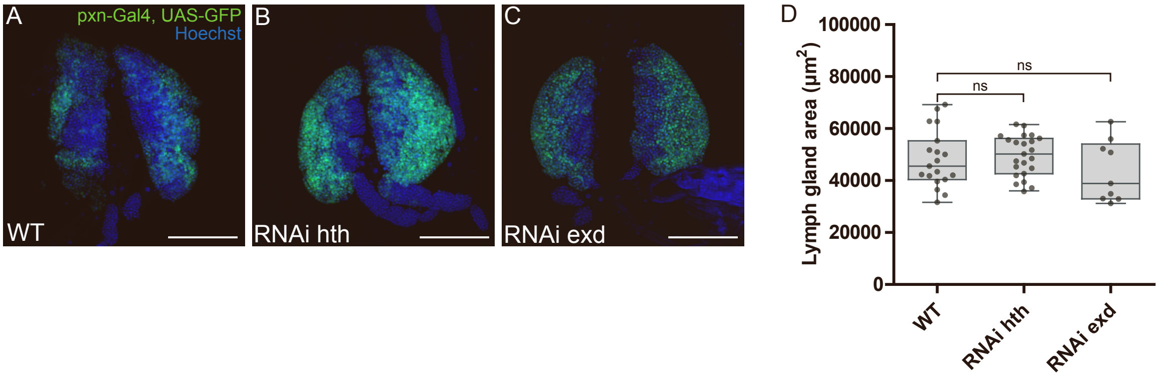

Figure S7: Depletion of hth or exd alone does not affect lymph gland size, related to Figure 6. **A-C**: Representative immunofluorescence confocal images of wL3 lymph glands. Cortical zone is marked by GFP expression and PSC is detected by immunostaining for antp (in red). Images are maximum intensity projection of Z-stacks. Scale bars 100 µm. **D:** Quantification of lymph gland area (μm^2) from immunofluorescence confocal images in A-C. Box plots show mean ± the 95th percentile as error bars. One-way ANOVA with Bonferroni post-test was performed to assess significant differences. ns: not significant. **Genotypes: A, D:** *pxn-Gal4, UAS-GFP/+* (WT) **B, D:** *pxn-Gal4, UAS-GFP/UAS-RNAi hth KK108831* **C, D:** *pxn-Gal4, UAS-GFP/UAS-RNAi exd KK107300*

Table S1. Fly lines, related to STAR Methods.

Genotype	Reference in manuscript	Provider	Stock number/reference
w, pxn-Gal4, UAS-GFP		F. Lemieux	[S1]
wiso	WT	M. Therrien	
w; UAS-MLL-AF4 ML5 (on 2nd)	MLL-AF4 (on 2nd)	R. Paro	[S2]
w; UAS-MLL-AF4 ML6 (on 3rd)	MLL-AF4 (on 3rd)	R. Paro	
w; UAS-MLL FL on 2nd		R. Paro	
w; UAS-MLL FL on 3rd		R. Paro	
w; UAS-MLL N-term (on 3rd)		R. Paro	
w; UAS-MLL N-term (on X)		R. Paro	
w; UAS-AF4 C-term on X		R. Paro	
UAS-RNAi ear TRiP.HMS00107}attP2	RNAi ear #2	Bloomington	34798
UAS-RNAi ear TRiP.JF02905}attP2	RNAi ear #1	Bloomington	28068
UAS-RNAi Su(Tpl) TRiP.GL00168}attP2		Bloomington	35270
UAS-RNAi Su(Tpl) TRiP.HMS00277}attP2	RNAi Su(Tpl)	Bloomington	33399
UAS-RNAi Mnn1 TRiP.HMC03436}attP40		Bloomington	51862
UAS-RNAi Mnn1 TRiP.JF01775}attP2		Bloomington	31220
UAS-RNAi Mnn1 TRiP.GL00018}attP2	RNAi Mnn1	Bloomington	35150
w[1118]; UAS-RNAi Mnn1 GD8117		VDRC	17701
UAS-RNAi Mnn1 KK101050		VDRC	110376
UAS-RNAI-CG7946 TRiP.HMC03961}attP40	RNAi Jasper	Bloomington	55274
y[1] v[1]; UAS-gpp-RNAi (TRiP.JF01284) (on 3rd)		Bloomington	31327
y[1] v[1]; UAS-gpp-RNAi (TRiP.JF01283) (on 3rd)	RNAi gpp #2	Bloomington	31481
UAS-RNAi gpp VDRC 47199		VDRC	47199
y[1] sc[*] v[1]; UAS-RNAi-gpp (TRiP.HMS00160) (on 3rd)	RNAi gpp #1	Bloomington	34842
y[1] sc[*] v[1]; UAS-gpp-RNAi (TRiP.GL01325) (on 3rd)		Bloomington	41893
y[1] sc[*] v[1]; UAS-gpp-RNAi (TRiP.HMS02612) (on 2nd)		Bloomington	42919
UAS-gpp-RNAi KK107875		VDRC	110264
gpp E-60/TM6b		M. Therrien	[S3]
gpp[03342]/TM6b		Bloomington	11585
y[1] v[1]; UAS-luciferase-RNAi TRiP.JF01355		Bloomington	31603
w[1118]; P{w[+mC]=UAS-lacZ.NZ)J312		Bloomington	3956
UAS-RNAi-lilli TRiP.JF02087}attP2		Bloomington	26314
UAS-RNAi-lilli TRiP.HMS01066}attP2		Bloomington	34592
UAS-hth-RNAi KK108831		VDRC	100630
UAS-exd-RNAi KK107300		VDRC	100687

Table S2. Primer sets used in qRT-PCR, related to STAR Methods.

Gene name	Forward primer	Reverse primer
MLL/KMT2A	CTCCTCTCTTCCCTTGGTTTAC	CTCTTGTCAGCATCTCGATCTT
AF4	CAGACTCCCATTGCCTTTGA	AGCAGGTCTAGGGTGATCTT
MLL-AF4	ACTCCTAGTGAGCCCAAGAA	CTTATTGACCGGAGGTGGTTT
Su(Tpl)	AAATGCCTGCCCAATGCACTA	CTTGCTATCATCGGTGTAACGG
gpp	TGGAAAAAGGAACTTCACTGCC	CTCGTACTGGTTGAGTTTATCCG
Mnn1	ATGTCCACGATTACCAGAAGCG	ACGGAACAGATTGATAACATCGG
ear	CTTTGTGCTGCACGAATCCTT	CAACAGGCAGTAGGAAGCCAG
Jasper	AACTAGAGAGCGGCCAAATC	GCCTCCTGCTTGGGTATTT
Rpl32	GCCCAAGGGTATCGACAACA	GCGCTTGTTCGATCCGTAAC
gapdh	TAAATTCGACTCGACTCACGGT	CTCCACCACATACTCGGCTC

Table S3. **q**RT-PCR results for all RNAi lines tested for knock-down efficiency in circulating hemocytes (hml-Gal4; UAS-GFP), related to STAR Methods.

Target	RNAi (or mutant) line	Mean relative expression of target gene (normalized to WT)	Standard deviation
Mnn1	UAS-RNAi Mnn1 TRiP.GL00018}attP2	0,2591	0,1082
Mnn1	UAS-RNAi Mnn1 TRiP.JF01775}attP2	0,9281	0,5574
Mnn1	UAS-RNAi Mnn1 TRiP.HMC03436}attP40	0,0829	0,0525
Mnn1	w[1118]; UAS-RNAi Mnn1 GD8117	0,7720	0,0396
Mnn1	UAS-RNAi Mnn1 KK101050	0,4792	0,1686
gpp	w[1118]; UAS-gpp-RNAi/CyO VDRC 47199	1,0582	0,2757
gpp	y[1] v[1]; UAS-gpp-RNAi (TRiP.JF01284) (on 3rd)	0,3778	0,1071
gpp	y[1] v[1]; UAS-gpp-RNAi (TRiP.JF01283) (on 3rd)	0,6124	0,2450
gpp	y[1] sc[*] v[1]; UAS-RNAi-gpp (TRiP.HMS00160) (on 3rd)	0,6864	0,1030
gpp	y[1] sc[*] v[1]; UAS-gpp-RNAi (TRiP.GL01325) (on 3rd)	0,8638	0,2574
gpp	y[1] sc[*] v[1]; UAS-gpp-RNAi (TRiP.HMS02612) (on 2nd)	0,6263	0,1941
gpp	UAS-gpp-RNAi VDRC KK110264	0,5784	0,1126
gpp	gpp E-60/TM6b	0,4222	0,1385
gpp	gpp[03342]/TM6b, Tb	0,2185	0,0483
Su(Tpl)	UAS-RNAi Su(Tpl) TRiP.GL00168}attP2/TM3, Sb[1]	1,1418	0,2144
Su(Tpl)	UAS-RNAi Su(Tpl) TRiP.HMS00277}attP2	0,4173	0,1145
ear	UAS-RNAi ear TRiP.JF02905}attP2	0,7164	0,6151
ear	UAS-RNAi ear TRiP.HMS00107}attP2	0,6065	0,2594
Jasper	UAS-RNAI-CG7946 TRiP.HMC03961}attP40	0,1049	0,0494
lilli	UAS-RNAi-lilli TRiP.JF02087}attP2	1,6282	0,3367
lilli	UAS-RNAi-lilli TRiP.HMS01066}attP2	0,9807	0,3801

SI References

S1. Stramer, B., Wood, W., Galko, M.J., Redd, M.J., Jacinto, A., Parkhurst, S.M., and Martin, P. (2005). Live imaging of wound inflammation in Drosophila embryos reveals key roles for small GTPases during in vivo cell migration. J Cell Biol *168*, 567-573. 10.1083/jcb.200405120.

S2. Muyrers-Chen, I., Rozovskaia, T., Lee, N., Kersey, J.H., Nakamura, T., Canaani, E., and Paro, R. (2004). Expression of leukemic MLL fusion proteins in Drosophila affects cell cycle control and chromosome morphology. Oncogene *23*, 8639-8648. 10.1038/sj.onc.1207904.

S3. Gavory, G., Baril, C., Laberge, G., Bidla, G., Koonpaew, S., Sonea, T., Sauvageau, G., and Therrien, M. (2021). A genetic screen in Drosophila uncovers the multifaceted properties of the NUP98-HOXA9 oncogene. PLoS Genet *17*, e1009730. 10.1371/journal.pgen.1009730.
